# Diagnosis and management of Cornelia de Lange syndrome: first international consensus statement

**DOI:** 10.1038/s41576-018-0031-0

**Published:** 2018-07-11

**Authors:** Antonie D. Kline, Joanna F. Moss, Angelo Selicorni, Anne-Marie Bisgaard, Matthew A. Deardorff, Peter M. Gillett, Stacey L. Ishman, Lynne M. Kerr, Alex V. Levin, Paul A. Mulder, Feliciano J. Ramos, Jolanta Wierzba, Paola Francesca Ajmone, David Axtell, Natalie Blagowidow, Anna Cereda, Antonella Costantino, Valerie Cormier-Daire, David FitzPatrick, Marco Grados, Laura Groves, Whitney Guthrie, Sylvia Huisman, Frank J. Kaiser, Gerritjan Koekkoek, Mary Levis, Milena Mariani, Joseph P. McCleery, Leonie A. Menke, Amy Metrena, Julia O’Connor, Chris Oliver, Juan Pie, Sigrid Piening, Carol J. Potter, Ana L. Quaglio, Egbert Redeker, David Richman, Claudia Rigamonti, Angell Shi, Zeynep Tümer, Ingrid D. C. Van Balkom, Raoul C. Hennekam

**Affiliations:** 1 Harvey Institute of Human Genetics, Greater Baltimore Medical Centre, Baltimore, MD USA; 2 Cerebra Centre for Neurodevelopmental Disorders, School of Psychology, University of Birmingham, Birmingham, UK; 3 Department of Paediatrics, Presidio S. Femro, ASST Lariana, Como, Italy; 4 Kennedy Centre, Department of Paediatrics and Adolescent Medicine, Rigshospitalet, Glostrup, Denmark; 5 Division of Human Genetics, Children’s Hospital of Philadelphia, and Department of Pediatrics, University of Pennsylvania Perelman School of Medicine, Philadelphia, PA USA; 6 GI Department, Royal Hospital for Sick Children, Edinburgh, Scotland UK; 7 Departments of Otolaryngology and Pulmonary Medicine, Cincinnati Children’s Hospital Medical Centre, University of Cincinnati, Cincinnati, OH USA; 8 Division of Pediatric Neurology, Department of Paediatrics, University of Utah Medical Centre, Salt Lake City, UT USA; 9 Paediatric Ophthalmology and Ocular Genetics, Wills Eye Hospital, Thomas Jefferson University, Philadelphia, PA USA; 10 Jonx Department of Youth Mental Health and Autism, Lentis Psychiatric Institute, Groningen, Netherlands; 11 Unit of Clinical Genetics, Paediatrics, University Clinic Hospital ‘Lozano Blesa’ CIBERER-GCV02 and ISS-Aragón, Department of Pharmacology-Physiology and Paediatrics, School of Medicine, University of Zaragoza, Zaragoza, Spain; 12 Department of Paediatrics, Haematology and Oncology, Department of General Nursery, Medical University of Gdansk, Gdansk, Poland; 13 Child and Adolescent Neuropsychiatric Unit, Fondazione IRCCS Cà Granda Ospedale Maggiore Policlinico, Milan, Italy; 14 CdLS Foundation UK and Ireland, The Tower, North Stifford, Grays, Essex UK; 15 Harvey Institute of Human Genetics, Greater Baltimore Medical Center, Baltimore, MD USA; 16 Department of Paediatrics, ASST Papa Giovanni XXIII, Bergamo, Italy; 17 Department of Genetics, INSERM UMR1163, Université Paris Descartes-Sorbonne Paris Cité, Hôpital Necker-Enfants Malades, Paris, France; 18 Human Genetics Unit, Medical and Developmental Genetics, University of Edinburgh Western General Hospital, Edinburgh, Scotland UK; 19 Division of Child and Adolescent Psychiatry, John Hopkins University School of Medicine, Baltimore, MD USA; 20 Centre for Autism Research, Children’s Hospital of Philadelphia, Philadelphia, PA USA; 21 Department of Paediatrics, Academic Medical Centre, University of Amsterdam, Amsterdam, Netherlands; 22 Section for Functional Genetics, Institute for Human Genetics, University of Lübeck, Lübeck, Germany; 23 CdLS World Federation’s, Hertogenbosch, Netherlands; 24 Wicomico County Board of Education, Salisbury, MD USA; 25 Clinical Paediatric Genetics Unit, Paediatrics Clinics, MBBM Foundation, S. Gerardo Hospital, Monza, Italy; 26 Danbury Public Schools, Danbury, CT USA; 27 Kennedy Krieger Institute, Johns Hopkins School of Medicine, Baltimore, MD USA; 28 Department of Gastroenterology, Nationwide Children’s, Columbus, OH USA; 29 Genética Médica, Hospital del Este, Eva Perón, Tucumán, Argentina; 30 Department of Clinical Genetics, Academic Medical Centre, University of Amsterdam, Amsterdam, Netherlands; 31 Department of Educational Psychology and Leadership, Texas Tech University, Lubbock, TX USA; 32 The Sidney Kimmel Medical College of Thomas Jefferson University, Philadelphia, PA USA; 33 Rob Giel Research Centre, Department of Psychiatry, University Medical Centre Groningen, Groningen, Netherlands

**Keywords:** Medical genetics, Clinical genetics, Disease genetics, Genetic counselling, Genetic testing, Signs and symptoms

## Abstract

Cornelia de Lange syndrome (CdLS) is an archetypical genetic syndrome that is characterized by intellectual disability, well-defined facial features, upper limb anomalies and atypical growth, among numerous other signs and symptoms. It is caused by variants in any one of seven genes, all of which have a structural or regulatory function in the cohesin complex. Although recent advances in next-generation sequencing have improved molecular diagnostics, marked heterogeneity exists in clinical and molecular diagnostic approaches and care practices worldwide. Here, we outline a series of recommendations that document the consensus of a group of international experts on clinical diagnostic criteria, both for classic CdLS and non-classic CdLS phenotypes, molecular investigations, long-term management and care planning.

## Abstract (web summary)

Cornelia de Lange syndrome is a genetic disorder affecting multiple organ systems that exhibits great phenotypic heterogeneity. This Consensus Statement summarizes recommendations for the diagnosis and management of patients with Cornelia de Lange syndrome.

## Introduction

Cornelia de Lange syndrome (CdLS) (Online Mendelian Inheritance in Man (OMIM) entries 122470, 300590, 300882, 610759 and 614701) is a multisystem disorder with physical, cognitive and behavioural characteristics that is named after the Dutch paediatrician Cornelia de Lange, who first described the developmental disorder in two infants in 1933 (ref.^1^). The prevalence is estimated to be between 1 in 10,000 and 1 in 30,000 live births^2^.

**Fig. 1:**
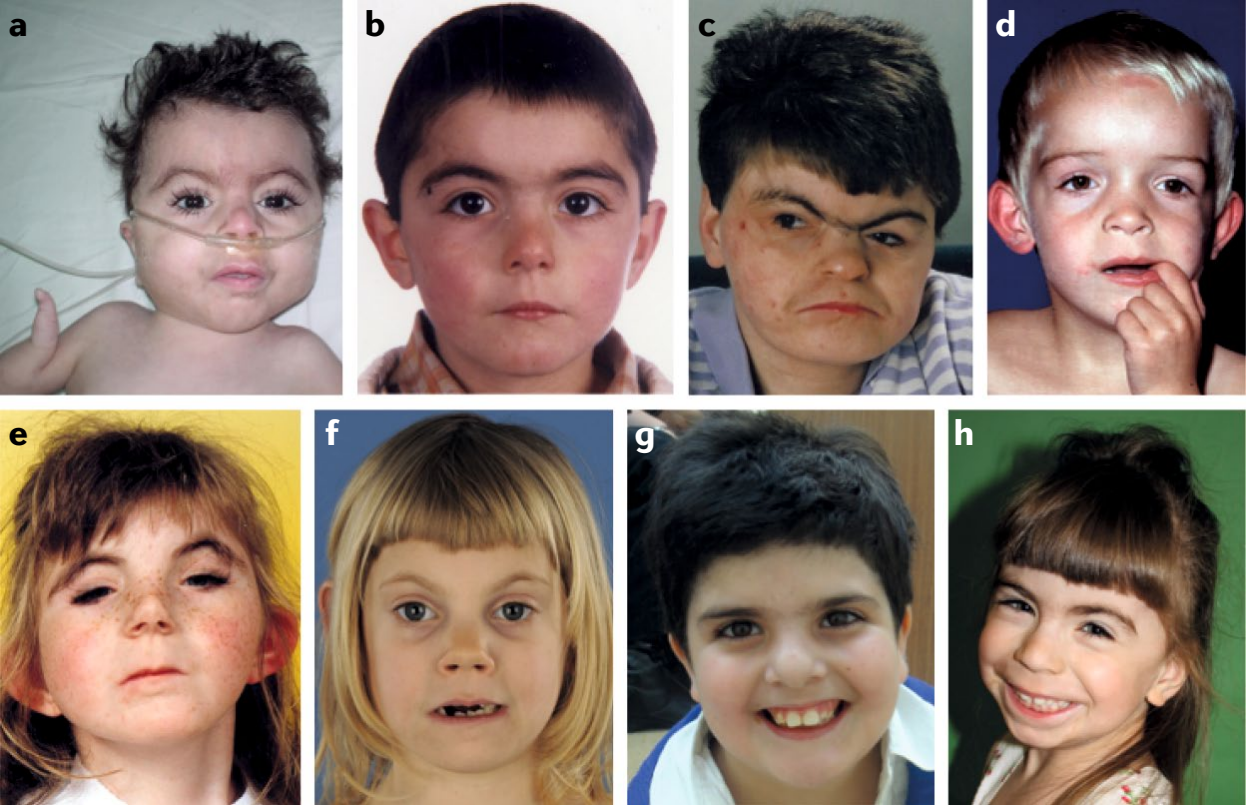
Facial phenotype of individuals with Cornelia de Lange syndrome. **a** | Classic Cornelia de Lange syndrome (CdLS) phenotype resulting from an *NIPBL* variant. **b** | Non-classic CdLS phenotype in an individual harbouring an *NIPBL* variant. **c** | Adult with the classic phenotype (*NIPBL* variant). **d** | Non-classic phenotype in individual with an *SMC1A* variant. **e** | Classic phenotype in an individual with an *SMC3* variant. **f** | Non-classic phenotype in an individual with a *RAD21* variant. **g** | Non-classic phenotype in an individual with an *HDAC8* variant. **h** | Non-classic phenotype in an individual with an *ANKRD11* variant.

Classic (or typical) CdLS is easily recognized from birth by experienced paediatricians and clinical geneticists owing to a distinctive craniofacial appearance and growth pattern, as well as limb malformations (Fig. 1). However, not all individuals with CdLS exhibit the classic phenotype, and presentation of the disorder can vary widely, from mild to severe and with different degrees of facial and limb involvement.

Over the past decade, genome-wide technologies, which can detect abnormal copy number and/or sequence variation, have emerged as a new first-line test for individuals with clinically significant developmental disorders. While molecular investigations have successfully attributed the aetiology of CdLS to genetic variants of structural or regulatory components of the cohesin complex, using genotype as the gold standard for diagnosis has led to problems in clinical studies of CdLS. For example, plausibly causal variants have been identified in a confirmed CdLS gene, *SMC1A*, in individuals who exhibit no features of CdLS but have characteristics resembling Rett syndrome^3^. As another example, plausibly causal variants were found in genes that had been associated previously with developmental disorders but not with CdLS, such as *ANKRD11* and *NAA10*, in individuals with features of CdLS^4,5^. Finally, gene variants whose products are considered to function in the cohesin complex were reported in individuals who do not exhibit the classic CdLS phenotype. Taken together, this heterogeneity in presentation and causal genes has made it increasingly difficult to determine which combination of characteristics should still be called CdLS^6^.

**Fig. 2:**
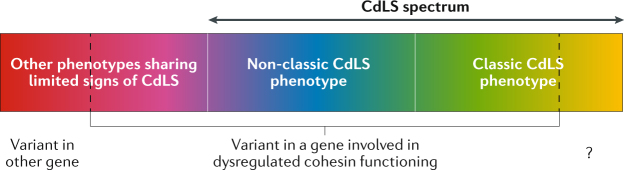
The phenotypes classified as Cornelia de Lange syndrome can be defined as a spectrum. The Cornelia de Lange syndrome (CdLS) spectrum includes individuals with the classic CdLS phenotype in whom a pathogenic variant in a gene involved in cohesin functioning has or has not been identified (if molecular confirmation is absent, the diagnosis can still be determined clinically), as well as individuals with a non-classic CdLS phenotype who harbour a pathogenic variant in a cohesin function-relevant gene. Individuals who carry a presumed pathogenic variant in a cohesin function-relevant gene but exhibit little or no resemblance to the classic CdLS phenotype do not fall within the CdLS spectrum. Please note that mildly affected and severely affected individuals may present both classic and non-classic CdLS. The question mark indicates that there may be genes causing CdLS spectrum that do not have a cohesin function; such genes are unknown at present, but their existence cannot be excluded.

The overall CdLS phenotype can be characterized as a spectrum (Fig. 2) to which the classic CdLS phenotype belongs as well as syndromes with a similar but non-classic phenotype caused by pathogenic variants in genes involved in cohesin functioning. A group of entities also caused by pathogenic variants in genes involved in cohesin functioning but showing only very limited overlap with the classic CdLS phenotype, such as Roberts syndrome (OMIM 268300) and Nicolaides–Baraitser syndrome (OMIM 601358), are not considered to be part of the CdLS spectrum. To date, there is no individual with a classic CdLS phenotype known to us in whom a variant in a gene without cohesin function has been reliably shown to be causative. All known causes of CdLS can thus be categorized as cohesinopathies, but not all cohesinopathies result in CdLS.

From the patient and family perspective, grouping individuals with a specific disorder facilitates knowledge exchange, establishes contact between affected individuals and their families who can support each other, and increases attention from scientists. However, indicating differences is also useful to tailor care to the individual. Recognizing the great phenotypic variability of CdLS and the wide heterogeneity in diagnostics, care and management of individuals with CdLS, a group of international experts, representing the Scientific Advisory Council of the World Federation of CdLS Support Groups, established an International CdLS Consensus Group to address these issues and define a series of recommendations that are presented in this Consensus Statement (for recommendations R1–R68, see Supplementary Box 1).

## Methods

The International CdLS Consensus Group comprised 43 participants from 30 institutions in 9 countries. The group consisted of clinicians, scientists and two patient-group representatives. The clinicians practice in North America, South America and Europe. A modified Delphi consensus process was adopted (Table 1). Discussions occurred via video conference calls, e-mail communications and file exchanges. All known support groups were contacted by e-mail to identify key issues that should be addressed during the consensus process. Subsequently, the issues to be addressed were determined by the Consensus Group in a video conference call. A plenary face-to-face 2-day meeting of 17 participants (including the patient-group representatives) was held in November 2017. Consensus recommendations were voted on by 37 participants (for recommendations R1–R68, see Supplementary Box 1).

**Table 1:** Details of the Delphi consensus voting process

Level of evidence	Definition	Votes (%)
+++	Evidence or general agreement indicate full agreement with the recommendation	≥70
++	Evidence or general agreement favour the recommendation	50–69
+	Evidence or general agreement are weak for the recommendation	26–49
–	Insufficient evidence or general agreement for the recommendation	<26

Voting was performed digitally by 37 co-authors of the guidelines. For all recommendations, >90% was in full agreement with the recommendations. Patient group representatives did not vote.

## Clinical diagnostic criteria

### Clinical features

**Fig. 3:**
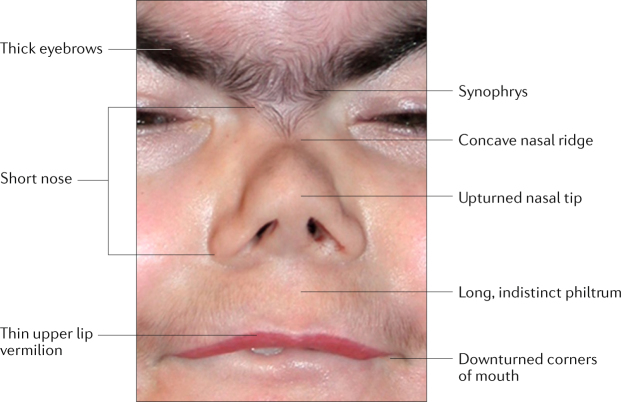
Cardinal facial features of Cornelia de Lange syndrome. Facial features that are the most characteristic for Cornelia de Lange syndrome (CdLS) include eye manifestations such as synophrys (meeting of the medial eyebrows in the midline) and thick eyebrows, a short nose, concave nasal ridge and upturned nasal tip, a long and smooth philtrum, a thin upper lip vermilion and downturned corners of the mouth. Non-facial features (not shown) that are considered to be cardinal features of CdLS include hand oligodactyly (the congenital absence of one or more fingers), adactyly (the absence of all fingers and/or toes) and congenital diaphragmatic hernia.

A combination of signs and symptoms defines the classic CdLS phenotype. We have classified these into cardinal features, considered to be the most characteristic for CdLS, and suggestive features, which add to the diagnosis but are less specific (Box 1; Fig. 3) (R1). We developed consensus criteria using these features: a score of ≥11 indicates classic CdLS if at least three cardinal features are present; a score of 9–10 indicates non-classic CdLS if at least two cardinal features are present; a score of ≥4 is sufficient to warrant molecular testing for CdLS if at least one cardinal feature is present; a score below <4 is insufficient to indicate such testing (R2). A score of ≥11 confirms the diagnosis of CdLS regardless of whether a pathogenic variant in one of the known genes can be found.

We tested the clinical diagnostic criteria in a series of 75 individuals with an *NIPBL* variant, 62 of whom had been scored in the past — independently of the present criteria — as having the classic phenotype, and 13 of whom were reported to have a non-classic phenotype^2,3^. Individuals with a classic phenotype and an *NIPBL* variant had a score between 12 and 16 (mean 13.5; cardinal features 3–5, mean 3.9), and those with the non-classic CdLS phenotype had a score between 9 and 11 (mean 10.0; cardinal features 2–3, mean 3.0). We also tested 46 individuals with an *SMC1A* variant, of whom 40 were identified as having a CdLS phenotype, and 6 were determined to have a phenotype resembling Rett syndrome^3^. Individuals with an *SMC1A* variant and a CdLS phenotype had a score between 2 and 13 (mean 8.0; cardinal features mean 2.9), and those with a Rett-like phenotype had a mean score of 3.5 (2–5; cardinal features mean 0.7). No individual with a CdLS phenotype and a molecular confirmation of a variant in *NIPBL* and *SMC1A* would have been missed using the presently proposed criteria. We tested the specificity of the clinical diagnostic criteria by applying them to a series of individuals with any of three entities that resemble CdLS (Coffin–Siris syndrome^7^, Rubinstein–Taybi syndrome^8^ or Nicolaides–Baraitser syndrome^9^); no individual scored as having a classic CdLS phenotype, and 4–10% scored as having a non-classic phenotype, indicating excellent discriminatory ability. The true specificity can be determined only in a separate, dedicated study.

Box 1 Clinical features of Cornelia de Lange syndrome **Cardinal features (2 points each if present)**

- Synophrys (HP:0000664) and/or thick eyebrows (HP:0000574)
- Short nose (HP:0003196), concave nasal ridge (HP:0011120) and/or upturned nasal tip (HP:0000463)
- Long (HP:0000343) and/or smooth philtrum (HP:0000319)
- Thin upper lip vermilion (HP:0000219) and/or downturned corners of mouth (HP:0002714)
- Hand oligodactyly (HP:0001180) and/or adactyly (HP:0009776)
- Congenital diaphragmatic hernia (HP:0000776)

**Suggestive features (1 point each if present)**

- Global developmental delay (HP:0001263) and/or intellectual disability (HP:0001249)
- Prenatal growth retardation (<2 SD) (HP:0001511)
- Postnatal growth retardation (<2 SD) (HP:0008897)
- Microcephaly (prenatally and/or postnatally) (HP:0000252)
- Small hands (HP:0200055) and/or feet (HP:0001773)
- Short fifth finger (HP:0009237)
- Hirsutism (HP:0001007)

**Clinical score**

- ≥11 points, of which at least 3 are cardinal: classic CdLS
- 9 or 10 points, of which at least 2 are cardinal: non-classic CdLS
- 4–8 points, of which at least 1 is cardinal: molecular testing for CdLS indicated
- <4 points: insufficient to indicate molecular testing for CdLS

Definitions according to Elements of Morphology. Human phenotype ontology identifier (HPO ID) numbers listed between brackets. CdLS, Cornelia de Lange syndrome.

### Severity scores

Several scoring procedures have been described to indicate the severity of CdLS^2,10–12^. No score takes the severity as experienced by the families into account, nor estimates the severity of all organ systems that may be affected in CdLS. We suggest that existing severity scores should be used with caution and acknowledge the need for the development of a severity score that represents severity as experienced by families, preferably stratified by genetic cause (R3).

## Molecular diagnostic criteria

### Genetics of CdLS

**Fig. 4:**
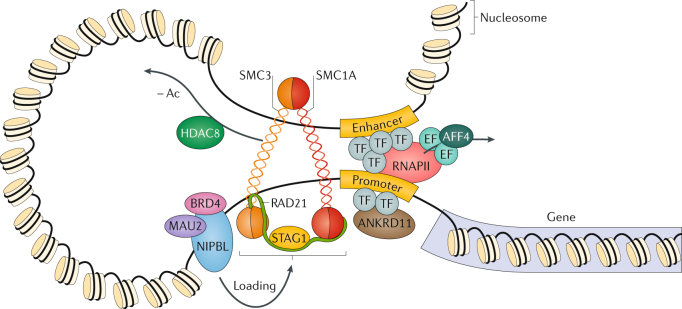
Cornelia de Lange syndrome is a cohesinopathy. Cornelia de Lange syndrome (CdLS) is caused by genetic variants that affect subunits or regulators of the cohesin complex. The structural core components double-strand break repair protein rad21 homologue (RAD21), structural maintenance of chromosomes protein 1A (SMC1A) and SMC3 of cohesin are thought to form a tripartite ring entrapping chromatids. In humans, cohesin subunit SA1 (STAG1), STAG2 or STAG3 directly attach to the ring and form part of the core complex. Nipped-B-like protein (NIPBL) and MAU2 chromatid cohesion factor homologue form a heterodimeric complex named kollerin that is required for cohesin loading onto DNA, and in which bromodomain-containing protein 4 (BRD4) interacts with NIPBL. Histone deacetylase 8 (HDAC8) regulates the cohesin complex release from chromatin by deacetylating SMC3. The functional interaction of ankyrin repeat domain-containing protein 11 (ANKRD11) with cohesin is under study but is currently unknown. Ac, acetyl group; AFF4, AF4/FMR2 family member 4; EF, elongation factor; RNAPII, RNA polymerase II; TF, transcription factor.

The CdLS spectrum has been associated with molecular abnormalities affecting genes involved in chromatin regulation, most commonly those involving the cohesin complex^13–18^ (Fig. 4). Cohesin is an essential regulator of most aspects of chromosome biology, including chromosome segregation, maintenance of genome stability, regulation of gene expression, chromatin structure and genome organization^19–22^. Although the exact pathomechanisms in CdLS are currently not fully understood, the direct role of cohesin as a regulator of gene expression is estimated to be of crucial importance for cohesin function.

In 2004, Nipped-B-like protein (NIPBL) was identified as the human homologue of the fungal and fly sister chromatid cohesion protein 2 (SCC2), which together with SCC4 forms a complex that is necessary for cohesin loading onto chromosomes, and variants in *NIPBL* were identified as the cause of classic CdLS^13,14^. Since then, cohorts of individuals with classic, non-classic and overlapping phenotypes have been screened for gene variants of other cohesin core and regulatory proteins, leading to the detection of variants in six additional genes causal of CdLS: *SMC1A*, *SMC3*, *RAD21*, *BRD4*, *HDAC8* and *ANKRD11* (R4). In a study in which 44 individuals were studied for all 5 genes known at the time to cause CdLS (*NIPBL*, *SMC1A*, *SMC3*, *RAD21* and *HDAC8*), and including a search for mosaicism, a causative variant was detected in 84% of individuals^23^. The clinical features of individuals with variants in the seven known genes differ in several aspects (Table 2).

**Table 2:** Comparison of the main clinical findings in individuals with molecularly confirmed Cornelia de Lange syndrome

	HPO ID*	*NIPBL*	*SMC1A*	*SMC3*	*BRD4*	*HDAC8*	*RAD21*	*ANKRD11*
*Growth*								
IUGR	0001511	+++	++	+	++	++	++	−
Short stature	0004322	+++	++	++	+	+	++	++
Microcephaly	0000252	++++	++	++	++	+	++	+
*Craniofacial features*								
Brachycephaly	0000248	++	+	+++	+	+++	++	+
Low anterior hairline	0000294	+++	+++	+++	++	++	+	+
Arched, thick eyebrows	0002253, 0000574	+++	+++	++++	+++	+++	+++	+
Synophrys	0000664	++++	+++	+++	+++	++++	+++	+
Long eyelashes	0000527	++++	+++	+++	+	+	+++	+
Depressed nasal bridge	0005280	+++	+	+	+	+	+	−^a^
Anteverted nostrils	0000463	+++	++	++	++	+++	+++	+
Broad nasal tip	0000455	++	++	+++	+	+	−	++
Long, smooth philtrum	0000343, 0000319	+++	++	++	++	++	++	++
Thin upper vermilion	0000219	++++	+++	+++	++	+	+++	++
Downturned corners of the mouth	0002714	++++	+++	++	+	++	+++	−
Highly arched palate	0000218	++	+	+	+	+	++	+
Widely spaced teeth	0000687	+++	+	+	−	++	−	−^b^
Micrognathia	0000347	+++	+	+	++	++	+	−
Low-set and malformed ears	0000369, 0000377	++	+	+	−	+	+	−
*Trunk and limbs*								
Oligodactyly and adactyly (hands)	0012165, 0009776	+	−	−	−	−	−	−
Small hands	0200055	+++	+++	+++	++	++++	+++	++
Proximally placed thumbs	0009623	++	+	+++	+++	+++	+	−
Clinodactyly or short fifth finger	0004209, 0009237	+++	+	++	+	++	+++	++
Small feet	0001773	++++	++	+++	NR	+++	+++	+
Hirsutism	0001007	+++	+++	++++	−	+	++	++
Cardiovascular anomalies	0002564	+	+	+	+	+	+	−
Vertebral anomalies	0003468	−	−	+	−	−	++	+++
*Cognition and behaviour*								
Intellectual disability (any degree)	0001249	++++	++++	++++	++++	++++	+	++++
ASD	0000729	+	+	+	−	+	+	+
Self-injurious behaviour	0100716	+++	+	NR	+	+	−	++
Stereotypic movements	0000733	++	++	NR	NR	−	−	−

ASD, autism spectrum disorder; HPO ID, Human Phenotype Ontology identifier; IUGR, intrauterine growth retardation; NR, not reported. *+*+++, ≥90%; +++, 70–89%; ++, 50–69%; +, 20–49%; −, <20%. ^a^Prominent nasal bridge. ^b^Macrodontia (larger than normal teeth).

#### NIPBL

Variants in *NIPBL* can be identified in approximately 70% of cases. Multiple studies have noted that loss-of-function variants cause more severe clinical features than missense variants, which are typically, albeit not always, associated with a less marked phenotype^10,11,24–26^. In addition to single nucleotide variants, microdeletions or intragenic exon deletions have been identified in 3% of cases^10,27,28^. Furthermore, a substantial number of individuals with classic CdLS carries mosaic *NIBPL* variants^23^. While individuals with the classic CdLS phenotype are likely to have variants in *NIPBL*, individuals with variants in one of the other causative CdLS genes can also fulfil the criteria for classic CdLS.

#### SMC1A

*SMC1A* encodes structural maintenance of chromosomes protein 1A, a core component of the cohesin complex (Fig. 4), and variants in this gene have been identified in an estimated 5% of individuals with CdLS^3^. Many individuals usually display a non-classic phenotype^3,15,16,24,29^ and have fuller eyebrows, a less striking shortening of the nasal bridge and a rounder face than individuals with *NIPBL* variants. A subset (40%) of individuals with *SMC1A* variants (as ascertained by panel sequencing for genes involved in intellectual disability) present with a phenotype distinct from CdLS that often resembles Rett syndrome^3,30,31^. *SMC1A* is an X-linked gene that is not inactivated^32^, and in the few families reported to date, female individuals are less affected than male individuals^3,15^. One parent, who showed no symptoms or signs of CdLS, was reported to harbour a mosaic *SMC1A* variant^16^.

#### SMC3

In 2007, a series of 115 individuals with CdLS was specifically investigated for variants in *SMC3*, which encodes another component of the cohesin complex (Fig. 4), and a single individual with atypical CdLS was found to have a variant in this gene^16^. *SMC3* variants are uncommon causes of CdLS^33^ and were also identified in individuals with intellectual disability, short stature and congenital anomalies who do not fulfil the clinical diagnostic criteria of non-classic CdLS^24,33^. *SMC3* variants identified in individuals with CdLS are typically missense changes^33^, suggesting that loss-of-function variants are not tolerated.

#### RAD21

Double-strand break repair protein rad21 homologue (RAD21) is another protein that is part of the cohesin complex^34^. To date, 13 individuals with *RAD21* variants have been reported, and our mutual experience adds 10 further variants, suggesting that *RAD21* variants comprise a small percentage of causes for CdLS. The first *RAD21* variants were reported in two individuals with a non-classic CdLS phenotype^17^. The subsequently reported individuals with a *RAD21* variant also had a non-classic phenotype^24,35^. Truncating and missense *RAD21* variants and intragenic deletions have been noted^36^, and missense variants were also reported in individuals without CdLS features^4,36^. The limited number of reported individuals precludes any genotype–phenotype correlation.

#### BRD4

Bromodomain-containing protein 4 (BRD4) is a chromatin-associated protein that localizes to clusters of enhancers by binding to acetylated histone H3 Lys27 (H3K27ac)^37^. The encoding gene was first implicated in CdLS when a de novo deletion that included *BRD4* was identified in an individual with an atypical CdLS phenotype; targeted sequencing subsequently determined de novo intragenic variants in *BRD4* (ref.^18^). Mass spectrometry identified NIPBL as a prevalent interacting protein in BRD4 immunoprecipitates, and missense variants were found to ablate the interaction with the acetylated histone while retaining the NIPBL association^18^, suggesting that sequestration of NIPBL underlies the pathogenic mechanism. The number of individuals with *BRD4* variants is too small to draw conclusions regarding the most common phenotype.

#### HDAC8

The first variants in *HDAC8* were reported in individuals with classic CdLS and in those with non-classic CdLS^38^ and in a family with X-linked intellectual disability and a phenotype that did not resemble CdLS^39^. To date, 65 individuals with *HDAC8* variants have been reported^24,38–42^. The variation in phenotype is remarkably wide and is typically non-classic, but some individuals fulfil the criteria for classic CdLS (Box 1). Distinctive features in affected individuals in addition to those of CdLS include a large anterior fontanel, widely spaced eyes (also known as orbital hypertelorism) and happy personalities. *HDAC8* is located on the X chromosome and can be inactivated^41^. Female carriers can be either affected or completely healthy, presumably depending on which X chromosome is inactivated. Most female individuals who are heterozygous for pathogenic *HDAC8* variants demonstrate marked skewing of X-inactivation towards the wild-type allele^41,42^.

#### ANKRD11

To date, five de novo *ANKRD11* variants have been reported in individuals with a non-classic CdLS phenotype^24,43^, and additional variants have been identified in clinical and research cohorts (unpublished observations, A.D.K., D.F., F.J.K. and R.C.H.). These patients have features that overlap with CdLS in facial gestalt, as well as some minor CdLS features (Box 1).

#### Other genes

In the search for further causes of CdLS, variants in several additional genes have been identified by exome sequencing; however, these variants were detected in individuals exhibiting limited clinical CdLS features rather than in individuals fulfilling the clinical diagnostic criteria for CdLS. De novo variants in *EP300* were detected in individuals with some features suggestive of CdLS^44^, and de novo *AFF4* variants have been reported in three individuals with CHOPS syndrome, which stands for cognitive impairment, coarse facies, heart defects, obesity, pulmonary involvement, short stature and skeletal dysplasia and includes features that overlap with CdLS^45^. Variants in *NAA10* have been described in a series of individuals with some resemblance to individuals with CdLS that is limited to the periorbital region^5^. Finally, recessive *TAF6* variants have been reported in two families with children who showed features that overlap with CdLS^4^.

### Mosaicism

Mosaicism has been found to occur frequently in CdLS^23^. Approximately 15–20% of individuals with classic features have mosaic *NIPBL* variants that cannot be detected in lymphocytes^23,24,46^. Rarely, individuals with CdLS can harbour mosaic *SMC3* (ref.^24^), *RAD21* (ref.^24^) or *SMC1A* variants (unpublished observations, D.F. and F.J.K.). It is assumed that mosaicism leads to variation in severity of the clinical phenotype but there is no formal proof of this at present. Selection against haematopoietic cells expressing variant *HDAC8* has been reported^41^, and the absence of *NIPBL* variants in blood cells but their presence in other tissues^23,46^ suggests that haematopoietic selection for expression of the normal allele occurs with *NIPBL* mosaicism. The gold standard for the identification of mosaicism is evaluation of DNA from uncultured fibroblasts, but circumstances may dictate the use of other tissues (R5). Analysis of DNA from buccal cell swabs, cultured fibroblasts, bladder epithelial cells, uncultured skin biopsy samples or surgical specimens has improved the detection rate for mosaic variants^23,24^.

### Familial recurrence risk

No large studies have been performed to determine gene-based familial recurrence risks in CdLS. Infrequently, families in which non-classic CdLS segregates in an autosomal dominant manner have been reported, as has germline mosaicism leading to affected siblings born to unaffected parents^47,48^. Our joint experience in 560 families including an individual with a causal *NIPBL* variant suggests that the familial recurrence risk owing to gonadal mosaicism is 0.89% (unpublished observations, A.D.K., M.A.D., F.J.R., J.W., V.C.-D., D.F., F.J.K., J.P., E.R. and R.C.H.). The recurrence risks for the X-linked *SMC1A* and *HDAC8* variants follow general rules of X-linked inheritance; by far, most have occurred as de novo events. If no molecular evaluation can be performed, the empirical recurrence risk is 1.5%^49^ (R6). Genetic counselling may be especially difficult for families in whom variants have been detected in *SMC1A*, *HDAC8* and *RAD21* owing to the remarkable variability of the phenotype, even within families^3,35,41,42^.

### Diagnostic approaches

#### Prenatal diagnostics

The major indications for prenatal diagnostics are an earlier child with CdLS, a new pregnancy in a family with a known genetic alteration in a CdLS gene or, as occurs most frequently, no family history but features suggestive of CdLS on fetal ultrasonography. In 73 published cases involving patients with prenatal findings suggestive of CdLS, symmetric intrauterine growth restriction (IUGR) with onset in the second trimester was noted as the most common finding (80%)^50^. Limb anomalies were seen in 66% of fetuses (likely representing a selection bias), and approximately 50% of fetuses had an abnormal facial profile (micrognathia and prominent maxilla)^51^. Other reported findings include increased nuchal thickness (51%), diaphragmatic hernia (28%) and cardiac malformation (15%)^50^. When considering prenatal investigations, the pros and cons of the prenatal studies need to be discussed with the parents to offer investigations tailored to their wishes and to the technical, medical and legal options available (R7).

Prenatal molecular testing can be performed on samples obtained from chorionic villous sampling or amniocentesis or by testing embryonic cells obtained through in vitro fertilization. Single-gene sequencing with or without deletion or duplication testing is used most frequently, but the advent of panel testing of chorionic villi or amniocytes allows assessment of all known causative genes in a single test in some countries (R8).

Non-invasive cell-free fetal DNA multi-gene screening that includes CdLS genes can identify de novo variants in families without a previous child who has CdLS. However, comparison with both biological parental samples is essential to interpret the large number of variants for which pathogenicity may be difficult or impossible to determine, which precludes meaningful use of this approach in routine practice at the present. Owing to the complexity of the molecular findings, prenatal testing for CdLS outside of a known familial pathogenic variant remains challenging. Interpretation of novel variants requires caution as pathogenicity may be difficult to determine, and the possibility of undetectable mosaicism often precludes using testing for exclusion purposes. For these reasons, the validity and informative value of prenatal test results, and the ethical issues these may raise for families in deciding whether to continue a pregnancy, must be considered and discussed with couples before sampling.

#### Molecular genetic testing

**Fig. 5:**
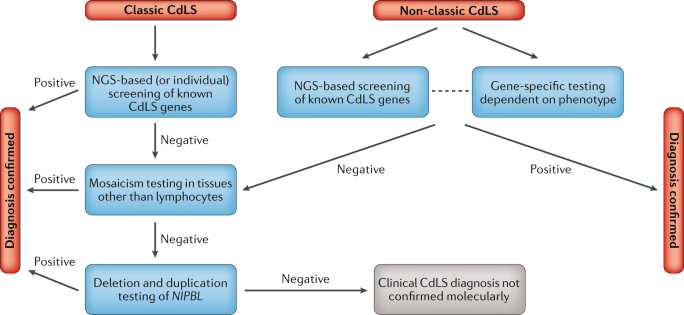
Molecular diagnostic pathways for Cornelia de Lange syndrome. In individuals with the classic Cornelia de Lange syndrome (CdLS) phenotype, the first-line molecular diagnostic approach should be next-generation sequencing (NGS)-based screening — either gene panel, whole-exome sequencing (WES) or whole-genome sequencing (WGS) — including currently known CdLS genes (*NIPBL*, *SMC1A*, *SMC3*, *RAD21*, *BRD4*, *HDAC8* and *ANKRD11*). If NGS is not available, molecular testing should begin with targeted sequencing of *NIPBL*. In individuals with the non-classic CdLS phenotype, the phenotype itself may allow experienced clinicians to determine which candidate gene should be sequenced first; if this cannot be determined, WES or WGS can be performed. In the case of negative results, *NIPBL* and subsequently the other CdLS genes should be tested for mosaicism using tissues other than blood, for example, fibroblasts, buccal swabs or bladder epithelial cells from urine. Deletion and duplication testing of *NIPBL* can be carried out using multiplex ligation-dependent probe amplification (MLPA) or chromosome microarray if first-line testing is not WES or WGS, through which deletions and duplications can be readily detected. If WES or WGS are used for first-line testing, the data can be investigated further for variants in other genes.

Panel sequencing is the most effective way of detecting causative variants in any of the genes known to cause CdLS, and first-line molecular testing should use a panel that contains at least the seven known CdLS genes (Fig. 5). Most diagnostic laboratories include several additional genes that can cause a phenotype resembling CdLS, such as *CREBBP* and *EP300*.

We realize that the availability of panel sequencing varies widely around the world, and financial constraints may dictate that clinicians work with other technologies. If panel sequencing is unavailable, Sanger sequencing of *NIPBL* in an individual with the classic phenotype would be the preferred approach for molecular testing. For individuals with non-classic phenotypes, evaluation of the phenotype may allow experienced clinicians to determine which of the other candidate genes should be sequenced first (R9).

If panel or Sanger sequencing does not detect causal variants, a study aimed at detecting mosaicism should be considered, preferably using uncultured fibroblasts, although buccal cells or bladder epithelial cells can also be used. If negative, testing for deletions or duplications of *NIPBL* using multiplex ligation-dependent probe amplification (MLPA) should be considered.

## Medical follow-up

### Paediatric medical follow-up

Given that CdLS can usually be recognized from birth, the paediatrician has a central role in clinical care. Once the clinical diagnosis of CdLS has been confirmed, every infant or child needs to be evaluated for common associated major malformations that require management or surveillance. Routine echocardiography and renal sonography are indicated in every diagnosed infant and child, given that 25% of individuals with CdLS have a cardiac anomaly and 10% have a renal malformation^49^. In adolescents, the usefulness of such studies should be guided by symptomatology (R10). Central nervous system imaging is indicated only if neurological symptoms such as seizures present, which is rare. Treatment and surveillance of major malformations are the same as for children without CdLS. In 50% of children with CdLS who have undergone intubation, the procedure has been difficult. Moreover, an adverse allergic reaction to midazolam (a short-acting benzodiazepine used for sedation) can occur^52^, although complications due to anaesthetic medications are rare^53^.

CdLS-specific growth charts are available^54^. Weight at birth is usually below the 5th percentile, and height, weight and head circumference all remain below the ranges for the general population (R11). The growth charts are derived from clinically diagnosed individuals, and no growth charts subdivided by molecular background are available. Growth is influenced by the nature of the variant and the causative gene^10,24^ and tends to be less compromised in individuals with *SMC1A* variants compared with those with *NIPBL* variants^3^. If growth velocity is lower than expected, gastrointestinal problems, thyroid dysfunction and growth hormone disturbances should be considered. Growth hormone secretion is normal in most children^55^, although a single child with a *NIPBL* variant with low growth hormone levels and an increase in growth after supplementation has been described^56^. The benefits of increased growth by growth hormone supplementation should be weighed against the burden of daily subcutaneous injections and the lack of a positive impact of an increased adult height on the quality of life for most individuals with CdLS.

Feeding difficulties are almost universally present in neonates and infants with CdLS and often in children and adults as well. Oral feeding is preferred if it is safe and stress-free and if feeding time does not exceed 3 hours per day, otherwise enteral feeding is recommended^57^. Involvement of dieticians is essential (R12, R13). Gastrostomies are the preferred option if tube feeding is needed for a prolonged period of time. Cleft palate, micrognathia and dental issues may contribute to feeding difficulties^57^. Cleft palate, including submucous cleft palate, occurs in 20% of individuals with CdLS. Isolated cleft lip is not related to CdLS. Dental problems consist of delayed secondary tooth eruption, small or absent teeth, malposition, malocclusion, overcrowding of teeth, dental caries on the perilingual maxillary surface (due to gastro-oesophageal reflux disease (GERD)), periodontal disease and bruxism. Dental problems are worsened by poor oral hygiene, especially in those with marked intellectual disability, and owing to limited patient compliance^58^ (R14, R15), which may lead to early-onset dental decay and periodontal disease^59^. Dental treatment by an interdisciplinary health-care team, a healthy diet, topical fluoride application and periodic dental check-ups are crucial in optimal management^60,61^.

Motor development is invariably delayed. Reliable data for a large series of individuals with molecularly confirmed diagnoses are not available. In a small series (*n = *51), children with *SMC1A* variants reached several milestones (sitting, walking and first words) at a younger age than children with *NIPBL* variants^3^. In the latter group, at 5 years of age, 99% were able to sit, 63% could walk independently and 38% had started to speak (R16).

Vaccinations should be given according to national schemes (R17). Recurrent respiratory infections are common and are thought to be secondary to altered anatomy, hypotonia and coordination of swallowing and coughing. Immunological anomalies occur occasionally; if unusually frequent or severe infections are present, further studies are indicated^62^. Thrombocytopenia has been reported but is usually non-progressive and asymptomatic^63,64^, and specific testing is not needed.

Pain can occur in children with CdLS, especially owing to dental problems, bladder and upper respiratory tract (including ears and sinuses) infections, gastro-oesophageal reflux and/or hip anomalies. Limited communicative abilities may hamper the shared recognition of pain^65^, and pain can lead to substantial behavioural problems. If there is suspicion that a patient with CdLS is in pain, the use of specific tools to identify pain in an individual with intellectual disability, such as the face, legs, activity, cry, consolability (FLACC) assessment tool, is recommended^66^ (R18).

Most individuals with CdLS will go through puberty. In clinically diagnosed individuals, puberty was mildly delayed (mean age of onset was 15 years for boys and 13 years for girls)^2^. That is, on average, menarche is delayed by 1 year compared with the general population; 5% of girls with CdLS will never menstruate. For those who do, the menstrual cycle often remains irregular. A bicornuate uterus is found in 19% of female patients, and approximately 80% of girls develop breast tissue. In boys with CdLS, 80% exhibit cryptorchidism, 37% have a small penis and 9% have hypospadias^2^. Surgical correction of cryptorchidism is recommended to reduce the risk of testicular cancer, as in the general population. No lowering of voice in boys at puberty has been reported^2^. Teenagers with CdLS can become overweight or develop overt obesity, which is often induced by high-calorie food offered by caregivers in combination with limited physical activity^2^; regular evaluation of weight is essential.

Preferably, all individuals with CdLS should be followed up by a paediatrician experienced in CdLS. Follow-up varies between countries but is frequent in infancy and early childhood, and annually to once every 3–5 years in adolescence and adulthood. In case of problems, the schedule should be adapted to include more frequent follow-up visits (R19).

### Adult medical follow-up

Currently, most people with CdLS reach adulthood owing to improved care, especially in the first year of life. Individuals with CdLS aged ≥50 years have been described^23,67^. Care coordination in adults is required, as many medical disciplines are typically involved.

A small number of women with CdLS have given birth, and often the diagnosis in the mother has been made only after diagnosis of the child^3,36,41,68^. A few men with CdLS are known to have fathered a child^69,70^, but reliable data on male fertility are not available. Sexual education should be offered appropriate to the level of socioemotional and cognitive functioning^71^ (R20). Contraceptive options are the same as for the general population. For some individuals, suppression of menses is preferred, and several contraceptives can effectively control or suppress menstruation. Hysterectomy is not recommended as a primary method of contraception but is sometimes employed for menorrhagia that does not respond to treatment^72^ (R21). Premenstrual syndrome and dysmenorrhoea occur in women with CdLS and can be associated with behavioural changes. Treatment options are as in the general population. We found no mention of menopause in CdLS in the literature, and no reliable studies on osteoporosis are available.

Several studies^49,59^ indicate that >30% of adults with CdLS are overweight, and at least 50% are considered obese. It remains uncertain whether this percentage is higher than in individuals with the same cognitive level and mobility. Obviously, caution with diet and physical activity is indicated (R22). Type 2 diabetes mellitus develops in 4% of individuals^2^.

Organ involvement is similar to that seen in the paediatric population and is discussed according to the major affected systems in detail below. Congenital heart anomalies should be detected in infancy or childhood and typically do not cause unexpected complications in adulthood. Hypertension and congestive heart failure have been reported in 4–8% and 2–4% of individuals with CdLS, respectively^2,73^. Fatal coronary occlusion and pulmonary artery embolism have been reported once^67^. In a retrospective cohort of 97 adults, 2 individuals had a myocardial infarction, and 2 had strokes^73^. Renal failure was reported in 1% of individuals but is rarely fatal^73^. Structural renal malformations were reported in 30% of adults; of these, 24% had abnormal creatinine clearance rates. It is recommended that renal function be monitored in those with structural renal malformations^64^ (R23). Prostate enlargement has been found in 10% of men by the age of 41 years, requiring prostate removal in one individual^74^. In the general population, benign prostatic hypertrophy is found in 25% of men in their fifties^75^, and international management recommendations, which can be followed for men with CdLS as well, start at age 45 years^76^ (R24).

Cancer of the oesophagus has been reported in three individuals with Barrett oesophagus. There is no increased risk of cancer at a young age, but reliable data for middle-aged and older individuals are not available. Screening for cervical and breast cancer should be performed according to standard guidelines^77,78^ (R25, R26).

In a study of 295 individuals with CdLS (81 infants, 117 children and 97 adults; 15 with a confirmed *NIPBL* variant), the most common causes of death in infants were congenital diaphragmatic hernia (17%) and respiratory problems (13%); in children, mortality was greatest owing to sequelae of congenital heart defects (10%) and respiratory (32%) and gastrointestinal problems (18%)^73^. No reliable data are available for the risk of death in infancy or childhood. Causes of death in adults are related to the gastrointestinal, pulmonary and cardiac systems, as well as to infections or to anaesthesia^2,67,73,79^. In several countries, individualized medical alert cards (also known as emergency cards) that report the main clinical data of the patient and the most frequent and potentially life-threatening medical complications of CdLS are used to the satisfaction of families and caregivers alike (Supplementary Boxes 2,3) (R27).

## Organ system manifestations

### Gastroenterology

Individuals with CdLS have more frequent gastrointestinal malformations, such as duodenal atresia, annular pancreas^80^, imperforate anus^81^, Meckel diverticulum^59^ and congenital diaphragmatic hernia^82,83^. Pyloric stenosis has been reported in up to 7% of patients^2,35^, and inguinal hernia is common in childhood^2^ (R28).

Intestinal malrotation has been reported in 5% of a series of 73 individuals^59^ and in 10% of a series of 49 individuals^2^, typically presenting as acute coecal volvulus. Intestinal malrotation may be recurrent^84,85^, may present in infancy, childhood or puberty as a surgical emergency, and may be difficult to diagnose owing to a lack of awareness by physicians, atypical symptoms and difficulties in effective communication with affected individuals (R29, R30). Coecal volvulus is associated with significant mortality^73^, which has led to the recommendation of imaging to prospectively check gastrointestinal mobility^85^. Rarely, sigmoid volvulus occurs^86^.

Constipation and rumination occur in 10–15% of adults with CdLS^2,73^ (in individuals with a *NIPBL* variant: 32%^2^; in those with *SMC1A* variants: 43%^3^; and in clinically diagnosed individuals with CdLS: 22–46%^2,59^). Individuals with constipation respond to treatment regimens similarly to the general population (R31). Diarrhoea (18%), gassiness (48%) and lactose intolerance (18%) are also fairly common^2^. There is no obvious increased association with coeliac disease^87^.

GERD is the most prevalent and severe gastrointestinal problem and can present in infancy as clinically significant dystonic Sandifer-like events^88,89^. GERD may become apparent in a highly variable manner, including feeding problems, recurrent (chemical) pneumonias, failure to thrive, agitation, restlessness or poor sleep. It has been suggested that self-injurious behaviour can be (partly) explained by GERD^3,90^ (R32). GERD tends to persist or to worsen with time. In a questionnaire study, GERD was more common in individuals with *NIPBL* variants (71%) than in those with *SMC1A* variants (60%)^3^. In a small series of 38 individuals with *NIPBL* variants, 55% had GERD^91^, and in a series of 43 clinically diagnosed individuals, the disease was more common in those with classic phenotypes (known to be caused predominantly by *NIPBL* variants)^92^. In this study group, aged 6–32 years, 65% of participants demonstrated inflammation of the lining of the oesophagus (oesophagitis) at endoscopy. In a study of 49 adolescents and adults with CdLS, 75% had GERD confirmed by gastrointestinal studies, and endoscopy demonstrated Barrett oesophagus in 10%, oesophageal metaplasia in 9%, eosinophilia in 16% and oesophageal narrowing (strictures) in 12%^2^. In another study, Barrett oesophagus was reported in 12% of individuals aged 6–32 years^93^. Mariani and co-workers^59^ reported clinical symptoms consistent with reflux in 71% of a group of individuals with partly molecularly confirmed CdLS, 38% of whom had endoscopy-confirmed oesophagitis. Several individuals with long-term GERD have developed oesophageal adenocarcinoma at a young adult age^92,94^. A strong correlation between Barrett oesophagus and oesophageal cancer is known in the general population^95^ and probably also applies to individuals with CdLS. Surveillance in those with Barrett oesophagus is widely recommended, although no randomized controlled trial is available that shows a more favourable course after surveillance; however, evidence suggests that it does improve outcome^95^.

No firm data are available on management results for GERD in larger series of individuals with CdLS. In clinical practice, a pragmatic trial with a proton pump inhibitor (PPI) in a child or adult with CdLS is preferred. Our mutual experience indicates that individuals with CdLS and GERD respond to PPIs at sufficiently high doses (omeprazole 0.7–3.5 mg per kg per day; for maintenance, usually half the dose is needed), similar to neurologically impaired individuals in general^57,96^. Modification of enteral nutrition and PPIs form the first-line treatment (R33). In case of a lack of response, an endoscopy should be considered (R34). Fundoplication and other surgical interventions are limited to those individuals with CdLS who fail to respond favourably to optimal nutritional and medical therapies (R33). Long-term follow-up is indicated as GERD is frequently chronic, which is a major risk factor for developing Barrett oesophagus^95^. Reliable surveillance can be performed only by repeated endoscopies, which puts a substantial burden on the individual with CdLS and their family, in particular owing to the anaesthesia that is needed for this procedure. In addition, we acknowledge that such diagnostic procedures differ in various countries for medicolegal and practical reasons. We suggest that the pros and cons of surveillance for Barrett oesophagus be carefully discussed with the family and, if possible, the individual with CdLS. Families and physicians should decide jointly what would be the optimal care for each person (R35); we therefore refrain from a general guideline on this aspect.

### Senses

#### Ophthalmology

Facial features are similar in adults and in younger individuals. Some individuals with CdLS have facial features that make them seem older than their chronological age, but reliable studies using 3D facial morphology are not available. Eye manifestations, such as synophrys (meeting of the medial eyebrows in the midline), thick eyebrows and long eyelashes are almost universally present in individuals with CdLS, regardless of the gene involved, and form one of the facial hallmarks of the syndrome. Ptosis — when the upper eyelid droops, obscuring part of the pupil — can occur unilaterally (37%) or bilaterally (44%), both in those with *NIPBL* variants and those without a molecularly confirmed diagnosis^26,97,98^. Surgery may be indicated, particularly when a compensatory chin lift is evident (present in 57%), which can interfere with ambulation, or when amblyopia (commonly known as lazy eye) or refractive error is thought to be secondary to the ptosis^97^ (R36). Blepharitis (25%) and related symptoms — epiphora, recurrent conjunctivitis, crusting on lashes, chalazion, corneal scars and opacities, and erythematous lid margins — can be bothersome, particularly for young children^97,99^. Unilateral or bilateral nasolacrimal duct obstruction occurs in 24% of individuals with *HDAC8* variants and in 67–80% of individuals with *NIPBL* variants^41^. Treatment of blepharitis is the same as in the general population: lid hygiene using baby shampoo or proprietary scrubs (R37). Surgical probing and irrigation for nasolacrimal duct obstruction should be considered only when symptoms are not improved with presumptive treatment for blepharitis^97^. Severe nasolacrimal duct obstruction may require nasolacrimal intubation and surgical correction (dacryocystorhinostomy)^97,98^.

Visual impairments occur in 44–53% of individuals with *NIPBL*, *SMC1A* and *HDAC8* variants^3,41^ and those without detectable disease-causing variants^98^. Spherical equivalent of >5.0  D (where D stands for dioptre) is seen in 38% of patients, and >10.0 D is seen in 9%^97^. Hyperopia (far-sightedness) is less common (15%), both in those with *NIPBL* variants and in those who had no molecular testing^98^. Astigmatism occurs both in molecularly confirmed and clinically diagnosed individuals.

As in all individuals with intellectual disabilities, regular assessment of vision is indicated (R38). Correction of refraction should be performed as early as possible to prevent amblyopia, although children often refuse glasses, and occlusion therapy may be difficult owing to an aversion to face touching. Contact lenses may be impractical for the same reason and because self-injurious behaviour may include hitting, pressing or poking of eyes. Surgical refractive procedures might improve visual function^100^.

Optic nerve abnormalities have been reported^98^. A peripapillary pigment ring can be found in up to 83% of individuals with CdLS^97^ as a benign finding without clinical consequences. Retinal detachment can occur owing to high myopia or self-induced trauma.

Nystagmus (rapid, involuntary eye movements), which is typically non-progressive, is found in 14–17% of patients^97,98^. Strabismus is found in 16–26% of individuals with CdLS, with esotropia occurring at a higher frequency than exotropia (61% versus 39%), and is slightly more prevalent in individuals with *NIPBL* variants (34.6%) than in individuals without molecular confirmation (21.4%)^97,98^. No specific studies are available on the management of strabismus in CdLS; strategies for the general population should be followed.

#### Ears, nose and throat

Individuals with CdLS typically have low-set, hairy and malformed ears, and one-third have small and stenotic ear canals^101^. Inspection of the eardrum using the common small paediatric speculum is frequently difficult, thus cerumen removal or middle ear evaluation may require sedation for a complete assessment.

Middle and inner ear abnormalities in individuals with CdLS include malformed ossicles, especially the malleus and incus, small mastoids, cochlear abnormalities, malformed vestibules and soft-tissue opacification of the tympanomastoid cavity^102,103^. Findings on temporal bone computed tomography, especially soft tissue in the middle ear, correlate well with audiometric data^103^, and imaging studies are useful to assess the cause of hearing loss.

Hearing loss is very common (85–90%) in individuals with CdLS^101,104,105^. It is typically bilateral, present in infancy, ranges from mild to severe (40–50%)^101^ and is sensorineural in 25% and conductive in 75%^104^ (R39). In adults, sensorineural hearing loss is reported in 45% of individuals with CdLS^105^.

Conductive hearing loss is often secondary to persistent otitis media with effusion (80–85%), and canal stenosis is present in 30% of individuals with CdLS^101,104,106^. Chronic or serous ear infection (otitis media) and chronic sinusitis are common (39%) in adulthood^107^ (R40). Initial evaluation of children with CdLS should include standard audiometric testing, plus otoacoustic emissions testing, auditory brainstem evoked response audiometry, or both^108^, to assess for auditory neuropathies^106^. Early identification of hearing loss is critical to maximize communication skills^101^. Hearing has been reported to improve with time in 50% of adults with CdLS, including those with severe hearing loss^106^. These findings indicate longitudinal evaluations of hearing.

Treatment options for hearing loss vary according to type and severity of the loss: in chronic or recurrent otitis media with effusion, myringotomy and pneumatic ear tube insertion are first-line treatments, and if soft tissue fills the middle ear and mastoid, mastoidectomy may be considered^102^ (R41). If pneumatic ear tube insertion is not effective, a standard hearing aid or a bone-anchored hearing aid are safe alternatives, but hearing aids can be poorly tolerated^109^. Cochlear implantation has resulted in variable levels of functional gain^110,111^. Surgical options, such as correction of an ossicular malformation, may be another option.

In individuals with CdLS, the nose is characterized by a low nasal bridge, short and concave nasal ridge and easily visible nares. One-third of individuals with CdLS experiences recurrent sinus infections, hypothesized to be caused by abnormal anatomy and disturbed humoral immunity^55^. Nasal polyps were reported^2^. Treatment of sinus infections should follow guidelines for the general population^112,113^. If an immune deficiency is present, more aggressive treatment, including immunoglobulins and prophylactic antibiotic treatment, may be indicated^62^.

Intubation can be difficult in individuals with CdLS, owing to a small mouth, small chin, short neck, stiffness of the temporomandibular joints and cleft palate^52,114^. Therefore, consultation with an anaesthesiologist before surgery is advisable (R42). Complications of anaesthesia in adults have been reported^53^.

### Orthopaedics

Children and adults with CdLS receive rehabilitation services across their lifespan. Adaptive equipment, such as orthotics, tripods, and wheelchairs, can markedly enhance motor functions and mobility, increasing quality of life. Moreover, safety equipment (for example, helmets, door alarms and seat belt harnesses) limits the risk of injuries and should be considered for every individual with CdLS.

Musculoskeletal problems are common, irrespective of the gene involved. In individuals with *NIPBL* variants, major upper limb anomalies are the most frequent (25%), whereas in those with variants in other genes, such malformations are infrequent (*SMC3*, *HDAC8* and *RAD21*) or absent (*SMC1A*)^3,115^. Individuals with truncating *NIPBL* variants have been reported to particularly have major limb defects^11,25,26,115^.

Major limb anomalies are almost exclusively found in the upper limbs, more frequently in male individuals^11^, and are asymmetric in 65% (in 75% of these individuals, the right side is the more affected side)^115,116^. Malformations include an absent forearm, abnormal fusion of the radius and ulna (radioulnar synostosis), absent radius or ulna, and oligodactyly. Polydactyly occurs only rarely. Small hands are present in almost all individuals with CdLS; radial head underdevelopment and radial dislocation are present in 79% of individuals^117^; other minor anomalies (proximally placed thumbs or abnormal curvature (clinodactyly) of the fifth fingers) are present in 65–85%^3,115–118^. Several studies in clinically diagnosed individuals with CdLS have suggested an association between major limb malformations and organ malformations, including diaphragmatic hernia and more marked intellectual disability, which likely can be explained by the more frequent presence of *NIPBL* variants in those with major limb malformations^3,25,115,118^.

In individuals with major anomalies, physical therapy or surgical procedures are usually not indicated as function is often remarkably good (R43). The use of full prosthetic devices has been attempted, but our joint experience indicates it is only rarely successful as the prostheses are not often accepted. The use of specific devices, for example, to allow independent eating, should be considered, as acceptance of such devices can be good (R44, R45). Minor limb anomalies do not require therapeutic interventions.

Major malformations of the lower extremities (absent tibia or fibula and split foot) are extremely uncommon^46,119^. Minor leg length differences occur in 46% of individuals, and Legg–Calvé–Perthes disease, a hip disorder that presents alongside disrupted blood flow to the femur, is seen in 4%^118^. Congenital hip dislocations are uncommon, and hip dislocations occur in 10% of individuals with CdLS later in life, especially in wheelchair-bound or bed-ridden individuals, as do tight hamstrings and Achilles tendons and contractures^59,115^. Management is as in the general population, taking the prognosis with respect to development and mobility into account. Preventive measures (physical therapy or orthoses) are paramount, and Botox injections and surgery may be indicated as well^120^. Minor lower limb anomalies (small feet, cutaneous syndactyly between the second and third toes, short fourth metatarsals or inward deviation of the big toes (hallux valgus)) are frequently present^3,10,59^. Specifically, deviation of the halluces may increase with age and cause walking difficulties^59^. In adults, 75% of individuals with CdLS report bunions, albeit without major complications; thus, surgical repair is not indicated^118^.

Scoliosis, especially thoracic scoliosis, develops in one-third of patients, often by 10 years of age^118^, and is more common in adults with decreased mobility^59^ (R46). Experience of scoliosis surgery is limited, but our mutual experience indicates that standard management is effective, taking prognosis with respect to development and mobility into account. Spine malformations are extremely rare and typically asymptomatic^121^, with kyphosis present in one-quarter of these individuals^118^. Flexion contractures have been reported in 18–25% of individuals with CdLS, particularly in the knees, which can interfere with ambulation, but also in the elbow and/or hip, as have tight Achilles tendons^118,122^.

### Neurology

Seizures are common in CdLS (individuals with *SMC1A* variants: 45%; with *NIPBL* variants: 15%; without molecular confirmation: 20–26%)^2,3,123^. Truncating *SMC1A* variants with marked seizures have been described in women with a Rett-syndrome-like disorder^3,31^ who do not fulfil the diagnostic criteria for CdLS^3^. In a series of 14 clinically diagnosed patients^124^ and an overview of individuals with mostly clinically diagnosed CdLS^123^, partial epilepsy was the most common type; age of onset was typically before 2 years of age, and 35 of 39 individuals for whom full data were available reacted well to standard therapy. Specifically, sodium valproate was found to be effective^124^ (R47). Anoxic epileptic seizures have been reported rarely^125^.

Isolated neurological signs, such as dystonia^126^, and catatonia^127^ are rare. Autonomic nervous system dysfunction, evaluated in a questionnaire study in 65 individuals with CdLS aged ≥8 years, was mildly disturbed in 81% of individuals and markedly disturbed in 26%^2^. Sensory deficits and temperature insensibility have been recognized by several of the present authors, but robust proof is lacking that this occurs more frequently in CdLS. The neuropathy may be linked to self-injurious behaviour^3^.

Structural brain abnormalities can occur, especially in individuals with *NIPBL* variants. NIPBL is known to regulate cortical neuron migration in mice^128^ and, indeed, cortical malformations have been described in individuals with CdLS caused by *NIPBL* variants^129^, as have small callosal bodies, white matter abnormalities, cerebellar anomalies and brainstem abnormalities. The limited number of available neuropathological reports confirms these brain anomalies^130^. In a small study of 15 clinically diagnosed individuals, no correlation between behaviour and magnetic resonance imaging (MRI) abnormalities could be detected^131^. MRI findings usually do not contribute to regular clinical care and should be limited to individuals with CdLS with neurological manifestations (R48). The presence of microcephaly in an individual with molecularly confirmed CdLS is in itself no indication for neuroimaging. Spinal cord abnormalities are rare and limited to (typically asymptomatic) tethered cord^129^. A single patient with a thoracic meningocele, a protrusion of the spinal cord through a bone defect in the vertebral column, has been reported^132^.

Sleep-related problems have been reported repeatedly, both in individuals with *NIPBL* and *SMC1A* variants and in clinically diagnosed individuals, with a widely variable frequency (12–72%) and ranging from nightly apnoea and insomnia to daytime drowsiness and frequent daytime napping^90,133–136^ (R49). Difficulties in falling asleep and staying asleep occur in 55–65% of individuals as early as infancy. Consecutive days without sleep were reported in 30% of 74 individuals with clinically diagnosed CdLS^135^. Snoring has been reported in 17% of 46 individuals with CdLS in one study^136^ and in 63% of 22 individuals with CdLS in another study^134^ and may lead to daytime sleepiness. Behavioural interventions and melatonin may be helpful in treating sleep disorders in those with CdLS (unpublished observations, L.M.K and A.Se.). Few adults have been reported to have sleep problems^135^, suggesting spontaneous improvements over time.

## Cognition and behaviour

### Cognition

Individuals with CdLS show intellectual disability that ranges from profound to mild; a small proportion performs within the normal range, whereas the majority has severe to moderate intellectual disability (Supplementary Tables 1,2). Individuals with *SMC1A* variants generally function at a higher level than those with *NIPBL* variants^3^. The type or site of *NIPBL* variants seems not to be associated with the level of intellectual disability^11,26,137^, although one study reported that missense and in-frame deletions were associated with less severe intellectual disability^138^.

Individuals with CdLS may have specific deficits in executive function beyond what is expected given the level of intellectual disability, especially in mental flexibility and visual short-term memory^139^. Assessment of the cognitive strengths and weaknesses of an individual and structuring their environment accordingly is beneficial. Comparative inhibition, another executive function, may be a relative strength^140^. Profiles of executive functioning may be associated with particular aspects of the CdLS behavioural phenotype, such as frequent repetitive behaviour or social anxiety^139,140^. Management strategies should include environmental enrichment strategies to stimulate cognitive and learning abilities.

### Sensory processing

Difficulties in sensory processing^141^ can lead to hyposensitivity and hypersensitivity, confusion and fixation by sensory stimuli and inconsistent sensation of stimuli^142,143^. Gastrointestinal and other somatic problems in individuals with CdLS may cause anxiety, mood disorders and challenging behaviour, including self-injurious behaviour^3,109,144^. No correlations with particular gene variants have been reported^145^, but our mutual clinical experiences suggest that all individuals with CdLS have sensory processing difficulties. Sensory processing difficulties are present across all levels of intellectual disability^146^; if an autism spectrum disorder (ASD) is also present, the behaviour can include low sensory thresholds^141^ and defensive responses towards sensory stimuli^147^. All interventions should address the sensory needs in order to enhance development and participation in daily living^146^ (R50).

### Adaptive behaviour profile

Individuals with CdLS demonstrate impaired adaptive behaviour across the lifespan, which is more marked in individuals with variants in *NIPBL* compared with other causes^148,149^. In CdLS, adaptive behaviour deficits are more marked than those with several other genetic conditions^150^, albeit comparable to Angelman syndrome and Rubinstein–Taybi syndrome^151–153^.

Adaptive behaviour skills in CdLS can decrease with age^10,154^. Changes over time can vary by specific skills, such as increased mastery with age of specific self-help skills (for example, washing and feeding) and decreased mastery of other skills (for example, ability to call for help or to move independently)^109^. Lower skill levels observed in older individuals with CdLS in cross-sectional studies may be confounded by cohort effects or recruitment biases, and true decreases in skills as individuals age remain uncertain. Indeed, mixed results have been reported^109,155^, which underlines the need for additional longitudinal research on adaptive behaviour skills (R51–R53).

### Self-injurious and aggressive behaviours

Self-injurious behaviour, that is, any self-directed behaviour that causes tissue damage, without intent of suicide or sexual arousal, such as self-hitting, head banging and self-biting, is frequent in individuals with CdLS^156^. Behaviours identical to self-injurious behaviour but not causing tissue damage may develop into self-injurious behaviour^157^. Risk markers for self-injurious behaviour include more severely compromised cognitive abilities, communication skills and adaptive behaviours, the presence of genetic variants in *NIPBL*, and increased levels of impulsivity, repetitive behaviours and characteristics commonly associated with diagnosis of ASD^156^. Clinically significant self-injurious behaviour occurs in 56% of individuals with CdLS, and hand-directed self-injurious behaviour is most common^158,159^. Physical injury in self-injurious behaviour can be scored on the basis of the amount of tissue damage and functional loss^156^. Restraints may be helpful to avert permanent tissue loss and have been used as first-line management^107^.

Self-injurious behaviour can be a sign of or response to pain. Careful medical evaluation is required, as self-injurious behaviour is associated with common medical conditions (GERD, otitis media, constipation, dental disease or hip problems). Best practices for the treatment of self-injurious behaviour require an integrated approach of medical evaluation, behavioural assessments and consideration of the environment. Treatment recommendations are then matched to the function of self-injurious behaviour (R54, R55).

There is limited evidence that self-injurious behaviour reacts to risperidone^160^, but this antipsychotic agent should only be used with careful monitoring of metabolic syndrome, weight gain and cognitive adverse effects. No data exist on the use of mood stabilizers in the management of individuals with CdLS.

### Repetitive behaviour

Repetitive behaviour, that is, behaviour linked by inflexibility, purposelessness, ritualization and (invariant) repetition, is characteristic of certain stages of typical infant development but may reappear with age in some disorders, including CdLS, and be aggravated by anxieties, sensory issues or social demands^161–163^. The frequency of repetitive behaviours is associated with more marked intellectual disability and with ASD^161,163^. In individuals with CdLS, repetitive behaviours can assume discrete manifestations, such as stereotyped motor movements, insistence on sameness and ritual behaviours, with lining up and tidying up being characteristic^162^.

In individuals with *NIPBL* variants, no correlation of repetitive behaviour with the site or nature of the variant was detected^10^. Studies employing longitudinal designs yielded contradictory results for changes over time^161,163^. Interventions should be sensitive to underlying issues, for example, anxiety, sensory problems or social demands, and consider environmental factors, such as predictability in daily structure. Psychopharmacology has centred on modulation of the serotonin pathway with selective serotonin re-uptake inhibitors (SSRIs) and the dopamine pathway with second-generation antipsychotic agents (SGAs). SSRIs are a standard of treatment for obsessive-compulsive disorder, but the effects of SSRIs in ASD are not convincing^164,165^. A cautious approach is indicated for the use of SSRIs in patients with CdLS because of possible behavioural activation and worsening agitation. SGAs may be useful for the management of rigidity and need for sameness, which can result in disruptive behaviours^160^.

### Social functioning including ASDs

Descriptions of common mental health issues in individuals with CdLS include ASD, (social) anxiety and mood disorders^109,137,152,154,165–169^, which do not seem to be associated with the site or nature of the gene variant involved^10,137,148,149,169^. Measuring psychopathology in CdLS is difficult because most affected individuals are unable to reliably report their own discomfort, behaviour or feelings. Symptoms commonly need to be inferred from proxy reports or observed behavioural presentations, such as eye-gaze avoidance, pushing away and screaming^80,170,171^. Maladaptive behaviours were positively correlated with parental stress levels^137^ and social context^109^.

The prevalence of ASD symptomatology in CdLS is 43%^159^. In individuals with *NIPBL* variants, ASD was associated with decreased adaptive behaviour skills^10^. ASD should be considered when social, communication and behavioural impairments are beyond what would be expected for the cognitive level of an individual.

The heightened prevalence of ASD symptomatology is not solely accounted for by associated degree of intellectual disability^150,172,173^. Comparisons of individuals with CdLS to those with ASD indicate broad similarities but subtle differences in specific areas of communication and social interaction^173^, especially social anxiety, extreme shyness and selective mutism^2,65,109,150,152^. The differences become more prominent with age and with increased social demand, and an individualized approach that is sensitive to these CdLS-specific aspects should make interventions more successful^65,166,174^. Rating scales and direct observational measures can provide a fine-grained evaluation of social functioning. Social motivation, social communication and enjoyment are substantially lower in those with CdLS in contrast to matched groups of individuals with other syndromes^151^ and are comparable to these traits in individuals with ASD^175^. This finding highlights the importance of detailed observations when evaluating ASD and social behaviour and of understanding the level and characteristics of communicative, adaptive and language abilities of those with CdLS. ASD-specific interventions may be helpful when used in conjunction with approaches that consider the broader social profile of the syndrome (R56–R58).

### Anxiety

Anxiety is common as a primary condition in individuals with CdLS, usually as generalized anxiety, separation anxiety^168^, selective mutism^148^ or as an exacerbating factor for repetitive behaviours, mood-related symptoms or disruptive, aggressive and self-injurious behaviours^109^. Anxiety may be difficult to assess, especially in individuals with challenging behaviours^137^. Social interactions can provoke anxiety and lead to observable behavioural responses, such as fidgeting, avoiding eye gaze and active avoidance of social interaction^152,175^. Individuals with CdLS have a heightened preference for sameness and have difficulties responding to changes in routine, which can make transitional periods more challenging and provoke anxiety^148,162,174^ (R59-R62).

### Communication and language

Communication abilities vary widely in individuals with CdLS but, typically, major difficulties are present, particularly in expressive language skills^170,176,177^, although well-developed speech and language can occur^138,174^.

Phonation, speech and mastication can be compromised in individuals with CdLS secondary to general abnormal muscle tone. Speech may also be impaired owing to hearing loss or morphological abnormalities of the palate, mandible and temporomandibular joint. Vision is also important for communication. Cognitive impairment can further complicate communication and understanding of communication^176,178^.

Individuals with CdLS tend to vocalize with a low-pitched cry and speak with a monotone voice^140,179^ regardless of cognitive level. Studies relating speech characteristics to genetic findings are lacking. There is a shortage of studies on the relationship between intellectual functioning, behaviour and communication abilities^169^. Selective mutism occurs as part of ASD or as an expression of anxiety^148,152^. In a small study of 17 individuals with CdLS, expressive language was found to be more compromised than receptive language, and receptive language compromised more than cognition, with specific deficits in morphosyntactic competences^138^. Expressive language is limited regardless of cognitive level.

In individuals with profound intellectual disabilities, communication is mostly determined by and dependent on sensitive responsiveness of the environment, and communicative signals are often subtle and easily overlooked^178^. Difficulties in social interaction and anxiety can worsen language skills^138^ and decrease intentional communicative behaviour compared with that of other individuals with a similar cognitive level but without difficulties in social interactions^170^. Challenging behaviours often co-occur with communication difficulties^3^ (R63).

Effective verbal and non-verbal communication skills can facilitate quality of life enormously, and speech therapy is highly recommended to optimize communication skills and should be implemented within the first 18 months of age (R64). Assessment of the level of communication of an individual and possible impediments are required for effective intervention of communication, as well as knowledge of suitable support tools^180^. Early augmentative and alternative communication interventions, tackling communication deficits from the beginning with specific attention to wide augmented communication input, are highly recommended^138,181,182^. Commonly used tools are gestures, icons, pictures and written language. Suitability differs by person, and a tailored approach is indicated.

Typically, parents are experts in understanding the communicative signals of their child. The experience they have acquired over the years is indispensable in cooperation with behavioural specialists and speech therapists. Detecting and identifying small communicative signals, awareness of one’s own reaction and understanding their meaning facilitates adjustment of communication and responses. Responsive milieu teaching and video observations can be very helpful in detecting and identifying such communication signals, their meanings and appropriate responses, especially in individuals with marked cognitive impairments^183,184^.

### Age-related changes in cognition and behaviour

Studies have reported age-related changes in behaviour, emotion and cognition^2,137,139,148,172^. In a study of 42 individuals with *NIPBL* variants, a (fairly weak) positive correlation was observed between chronological age and behavioural difficulties^137^, and statistically significant correlations were found between chronological age and measures of interest and pleasure, and insistence on sameness^148^, indicating older individuals exhibit more difficulties. Studies of clinically diagnosed individuals with CdLS have reported changes in verbal working memory, ASD symptomatology, anxiety, low mood, self-injurious behaviour and impulsivity^2,90,109,139,154,166,172,174^, indicating increasing difficulties in frequency and severity with age. Similar changes were not found for aggression, hyperactivity or sleep difficulties. The associations with age have been documented through correlation analyses. Studies using age-band analyses have identified that adolescence and early adulthood are periods of marked change^109,167,172^. Management strategies should include evaluation of social environment, support during transitional periods using a person-centred approach and gradual introduction of changes. Coordination of the support for the individual with CdLS by a named caregiver is helpful (R65).

## Care planning

### Medical care

A diagnosis of CdLS necessitates lifelong medical, multidisciplinary and social care, regardless of the molecular aetiology. Access to clinical evaluation, counselling and follow-up by a multidisciplinary team is likely to improve health care and increase quality of life. Recognized barriers to accessing care include health or behavioural complications, geographical isolation and financial considerations^185–187^. Individuals with CdLS are now likely to live into adulthood and old age and thus risk developing common chronic diseases in addition to CdLS-related morbidities. They are more likely to experience delayed treatment, to be hospitalized and to have more complications and longer admissions than individuals without CdLS owing to lack of knowledge regarding CdLS by caregivers, difficulties in obtaining a reliable earlier history and, possibly, stigmatization. The use of syndrome-specific, individualized treatment plans, including regular health checks, planning of admissions and discharges in advance, and the use of procedure-specific information booklets using simple language and photos are recommended^188,189^ (R66, R67).

### Transition

Transition of medical care from a paediatric to an adult care team comes with substantial challenges^190^ and requires parental involvement. Typically, transition coincides with changes in daytime environment, leaving home and legally mandated social changes. Care changes from family-focused to affected-individual-focused. Transitions that are initiated too late can result in a gap in communication and coordination between paediatric and adult services. Parents have suggested that care may be improved by establishing joint child and adult care clinics^109,191,192^ (R68).

### Decision-making

The involvement of individuals with CdLS and their care providers in health-care decisions is essential. A marked degree of intellectual disability and executive functioning in individuals with CdLS can decrease decision-making capacities; if so, care providers and health-care professionals have to decide what is best and document values, preferences and quality of life^193^. Knowledge about CdLS is essential to manage expectations, and health-care providers and social services need to be aware of the needs and problems that can be expected. Family support groups and social media have proved to be extremely helpful in this respect^194,195^. Guardianship rules vary among countries, and it is essential for guardianship to be determined and assigned before the age of majority.

## Conclusions

The present recommendations provide a framework for improving diagnosis and management of CdLS. CdLS is a complex disorder, in which many body systems are affected, and it is important that a lead clinician is identified for each patient to ensure coordination of the numerous aspects of care in both childhood and adulthood. The proposed clinical and molecular diagnostic pathways are intended to be universally practical, both in countries with access to modern techniques and in those where this is not yet possible, and they are meant to be cost-effective, avoiding unnecessary diagnostic or management procedures. Still, in some health-care systems and medicolegal environments, the guidelines may need to be adapted. It is important that implementation of the consensus recommendations is accompanied by prospective audits in order to expand the evidence base and allow for future ameliorations of the consensus.

## Supplementary information

Supplementary information

## Glossary

Phenotype: All morphological and functional attributes of an individual or of the organs, tissues or cells of that individual, except for the primary morphology of the genome.
Cohesin complex: A multisubunit protein complex that mediates sister chromatid cohesion and cellular long-distance chromatin interactions. In vertebrates, the cohesin complex is composed of structural maintenance of chromosomes protein 1A (SMC1A), SMC3, RAD21 and cohesin subunit SA1 (also known as STAG1).
Genotype: The primary DNA sequence, either overall or at a specific locus, of an individual or of the organs, tissues or cells of that individual.
Rett syndrome: A severe, progressive neurological disorder primarily affecting females that is mainly caused by mutations in the gene encoding methyl-CpG-binding protein 2 (MECP2). It is characterized by loss of acquired speech and motor skills, repetitive hand movements, breathing irregularities and seizures.
Cohesinopathies: Disturbances of the function of the cohesin complex that lead to altered human development.
Delphi consensus process: A structured communication process between a panel of experts.
Mosaicism: The presence of two or more populations of cells with different genotypes in a single individual, who has developed from a single fertilized egg cell.
Facial gestalt: The unified pattern of elements of facial characteristics that cannot be derived from the simple summation of its elements.
Cell-free fetal DNA: Circulating DNA in maternal plasma originating from the fetus.
Multiplex ligation-dependent probe amplification: (MLPA). A molecular technique involving the ligation of two adjacent annealing oligonucleotides followed by quantitative PCR amplification of the ligated products, allowing the characterization of chromosomal aberrations in copy number or sequence and single nucleotide polymorphism or mutation detection.
Chalazion: Small swelling of the eyelid due to a blocked meibomian gland.
Strabismus: Strabismus (or squint or ‘crossed eyes’) is non-parallel orientation of the visual axes of the eyes causing a disturbed vision, which can be inwards (esotropia) or outwards (exotropia).
