# Supplementary material for: Diagnosis and management of Cornelia de Lange syndrome: first international consensus statement
Source: Nat Rev Genet. 2018 Jul 11;19(10):649–66. doi: 10.1038/s41576-018-0031-0 (PMC7136165; doi:10.1038/s41576-018-0031-0)
Supplement: Supplementary file 1 — Supplementary information [file 41576_2018_31_MOESM1_ESM.pdf]

nature REVIEWS GENETICS



# Diagnosis and management of Cornelia de Lange syndrome: first international consensus statement

*Antonie D. Kline, Joanna F. Moss, Angelo Selicorni, Anne-Marie Bisgaard, Matthew A. Deardorff, Peter M. Gillett, Stacey L. Ishman, Lynne M. Kerr, Alex V. Levin, Paul A. Mulder, Feliciano J. Ramos, Jolanta Wierzba, Paola Francesca Ajmone, David Axtell, Natalie Blagowidow, Anna Cereda, Antonella Costantino, Valerie Cormier-Daire, David FitzPatrick, Marco Grados, Laura Groves, Whitney Guthrie, Sylvia Huisman, Frank J. Kaiser, Gerritjan Koekkoek, Mary Levis, Milena Mariani, Joseph P. McCleery, Leonie A. Menke, Amy Metrena, Julia O'Connor, Chris Oliver, Juan Pie, Sigrid Piening, Carol J. Potter, Ana L. Quaglio, Egbert Redeker, David Richman, Claudia Rigamonti, Angell Shi, Zeynep Tümer, Ingrid D. C. Van Balkom and Raoul C. Hennekam*

https://doi.org/10.1038/s41576-018-0031-0



© 2018 Macmillan Publishers Limited, part of Springer Nature. All rights reserved.

**Supplementary Box 1. Recommendations CdLS guidelines**

**R1:** Cornelia de Lange syndrome (CdLS) spectrum encompasses a range of phenotypes consisting of classic (or typical) CdLS and non-classic CdLS, which are characterized by a combination of features (Table 1).

**R2:** We propose consensus criteria based on the presence of a combination of signs and features (Table 1). A diagnosis of classic CdLS can be confirmed if a score of 11 is reached, irrespective of the presence of a causative variant in a gene known to result in CdLS.

**R3:** Presently available severity scoring schemes should be used cautiously as these do not adequately reflect the severity as experienced by the individuals with CdLS and their families.

**R4:** Classic CdLS is usually caused by variants in *NIPBL*; however, variants in one of six other genes — *SMC1A, SMC3, RAD21, BRD4, HDAC8* or *ANKRD11* — should be considered, as they may lead to a similar phenotype.

**R5:** Mosaicism should be considered in individuals with CdLS in whom a causative variant cannot be detected in lymphocytes, in which case other tissues such as fibroblasts, buccal cells or bladder epithelial cells should be studied.

**R6:** Genetic counselling should be offered to all families with a family member with CdLS. Families should be counselled that the recurrence risk of CdLS differs depending on the gene involved. In the non-X-linked forms, the recurrence risk is 0.9% due to germline mosaicism. Autosomal dominant inheritance of CdLS does occur. In clinically diagnosed individuals with CdLS, the empirical recurrence risk is 1.5%.

**R7:** If prenatal sonography detects features consistent with CdLS, possibilities for prenatal molecular studies should be discussed with the parents.

**R8:** If a causative variant has been detected in an earlier child or pregnancy, reliable prenatal diagnostic testing should be discussed with the family. Targeted variant testing can be performed using DNA derived from chorionic villi or amniocytes.

**R9:** If available, first-line molecular testing is performed using next-generation sequencing-based technology to screen all genes known to cause CdLS spectrum (*NIPBL, SMC1A, SMC3, RAD21, BRD4, HDAC8* and *ANKRD11*). Medico-legal, technical, and insurance-related national practices may require other tests, such as Sanger sequencing of individual genes.

**R10:** Every infant and young child with CdLS should be assessed for cardiac and renal malformations subsequent to diagnosis.

**R11:** The growth of every child with CdLS should be monitored by using CdLS-specific growth charts.

**R12:** In every CdLS individual with prolonged and marked feeding difficulties, the multidisciplinary assessment should consider (temporary) placement of a gastrostomy as a supplement to oral feeding.

**R13:** In individuals with CdLS who have recurrent respiratory infections, reflux and/or aspiration should be ruled out.

**R14:** The palate should be closely examined by both inspection and palpation at diagnosis. In case of symptoms of a (submucous) cleft palate, referral for specialist assessment is indicated.

**R15:** Dental assessment and cleaning should take place regularly; a more thorough dental examination or treatment under anaesthesia may be necessary.

**R16**: Developmental milestones should be closely monitored.

**R17:** Vaccinations should be given to every child with CdLS according to national guidelines.

**R18:** As pain can easily remain unrecognized in a child with CdLS, all care providers should be aware of the different manifestations and the possible sources of pain. Specific tools to assess pain are recommended.

**R19:** Regular follow-up of every child with CdLS is needed, preferably by a paediatrician experienced in treating individuals with CdLS; schedules depend on local practices and possibilities.

**R20:** Sexual education appropriate to the level of understanding should be offered, and contraception management should follow local standards for the general population.

**R21:** Hysterectomy is indicated if severe menorrhagia is present and does not respond to medical treatment.

**R22:** Specific attention to diet and stimulation of activities are recommended as overweight and obesity can occur.

**R23:** Renal function should be regularly monitored in children and adults with CdLS who have structural renal malformations.

**R24:** Prostate enlargement in men with CdLS should be treated according to national guidelines for the general population.

**R25:** Women with CdLS should be offered breast cancer screening according to national guidelines for the general population.

**R26:** Routine gynaecologic care including cervical screening should be performed in women with CdLS, according to national guidelines for the general population.

**R27:** The use of emergency cards should be considered for every person with CdLS.

**R28:** Every newborn suspected or proven to have CdLS should be carefully evaluated for signs and symptoms consistent with congenital gastrointestinal malformations.

**R29:** Any acute abdominal presentation in an individual with CdLS, irrespective of age, should prompt consideration of intestinal malrotation.

**R30:** Evaluation for the presence of intestinal malrotation needs to be discussed and decided together with the family, balancing the potential gain in health and burden for the individual with CdLS.

**R31:** Constipation is present in almost half of all individuals with CdLS and should be treated as in the general population.

**R32:** Consider always gastro-oesophageal reflux disease (GORD) in any individual with CdLS owing to its frequency and wide variability in presentation, which includes challenging behaviour.

**R33:** Modification of nutrition and proton pump inhibitors (PPI) are the first-line treatments of GORD. Anti-reflux medications need to be used to their maximum dosage. Surgical interventions for GORD should be limited to those individuals with CdLS in whom nutritional and medical treatments have been unsuccessful or airway safety is at risk.

**R34:** If GORD symptoms persist, endoscopy should be strongly considered whilst an individual with CdLS is still in paediatric care.

**R35:** Surveillance for Barrett oesophagus needs to be discussed with and decided together with the family, balancing the potential gain in health and burden for the individual with CdLS.

**R36:** Surgical correction of ptosis should be considered if vision is significantly affected or a compensatory chin lift is present that interferes with ambulation.

**R37:** Blepharitis in individuals with CdLS should be treated conservatively with lid hygiene. Nasolacrimal duct obstruction should be suspected if symptoms are not improved with lid hygiene.

**R38:** Vision should be regularly evaluated in all individuals with CdLS, especially in infancy and childhood. Refractive errors should be corrected early to prevent amblyopia, although children may have difficulty tolerating glasses or contact lenses.

**R39:** Hearing should be assessed in individuals with CdLS at an early age and should be followed longitudinally. Those with severe sensorineural hearing loss should be assessed for auditory neuropathy.

**R40:** Regular ophthalmological and otolaryngological evaluations are recommended in adults with CdLS.

**R41:** Otitis media with effusion and sinusitis in individuals with CdLS should be considered and treated according to the national guidelines for the general population.

**R42:** The anaesthesiologist should be aware of the potential difficulty with intubation in individuals with CdLS

**R43:** As function is often remarkably good in major limb anomalies, caution is recommended regarding orthopaedic procedures in individuals with CdLS.

**R44:** Prosthetic devices targeting a single function should be considered depending on the needs and tolerance in individuals with CdLS.

**R45:** Prognosis regarding development and mobility should be taken into account when considering treatment of orthopaedic problems in individuals with CdLS.

**R46:** Scoliosis and leg length differences need specific attention in adults with CdLS at regular medical check-ups.

**R47:** Seizures in individuals with CdLS should be treated using the general management schemes.

**R48:** An MRI of the brain should be considered only if the individual with CdLS shows neurological signs other than microcephaly.

**R49:** Sleep problems in individuals with CdLS can have serious consequences, and behavioural sleep management should be considered.

**R50:** Hyper- and hyposensitivity and other sensory processing difficulties should be assessed and support strategies should be implemented in individuals with CdLS throughout their lifespan.

**R51:** Increasing adaptive skills to enhance independence should remain a focus throughout the lifespan of individuals with CdLS and should include personalized specific goals and teaching strategies.

**R52:** Additional developmental and educational support should be provided to individuals with CdLS to reach their maximum cognitive and educational potential, taking into account their specific cognitive impairments.

**R53:** Cognitive strengths and weaknesses of individuals with CdLS should be assessed in order to design educational and interventional strategies.

**R54:** To identify the cause of self-injurious behaviour in individuals with CdLS, medical assessment, specifically looking for sources of pain, should be followed by behavioural assessment of self-restraint and then functional analysis.

**R55:** Treatment of self-injurious behaviour should include both medical and behavioural strategies.

**R56:** A clinical diagnosis of autism spectrum disorder (ASD) should be considered in all individuals with CdLS throughout life, taking into account atypical presentations.

**R57:** In addition to standardized ASD diagnostic tools, fine-grained observations should be carried out to accurately define the profile of social functioning in an individual with CdLS.

**R58:** ASD-specific interventions should be considered in all individuals with CdLS in conjunction with approaches that consider the broader social functioning profile of the syndrome.

**R59:** Interventions targeting problematic repetitive behaviour in individuals with CdLS should be sensitive to anxiety, sensory problems and social demands. These interventions should also consider environmental factors.

**R60:** Atypical presentation of anxiety and mood disorder should be considered when behavioural changes occur.

**R61:** As anxiety is common in individuals with CdLS during periods of environmental change, a planned program should be implemented.

**R62:** Treatment of anxiety and mood disorders in individuals with CdLS should be considered using psychosocial interventions and pharmacotherapy.

**R63:** When assessing communication, vision and hearing problems, speech impairments, intellectual disability, difficulties in social interaction and social anxiety should be considered. Video observations can be very useful.

**R64:** Developmentally appropriate communication strategies (such as speech therapy, augmented communication input) should be implemented within the first 18 months of life.

**R65:** Individuals with CdLS should receive extra support during adolescence and early adulthood, using a person-centred approach to mitigate mental health issues and problematic behaviour.

**R66:** Individuals with CdLS and their families need life-long care provided by healthcare providers and social services, who should educate themselves about CdLS.

**R67:** Syndrome-specific and personalized care plans through shared decision-making should be offered to every individual with CdLS and their care givers.

**R68:** Transition of care should be initiated at an early phase, with proper transfer of medical history and knowledge about the personal characteristics of the individual with CdLS. It is recommended that current and future health care providers jointly evaluate individuals with CdLS in order to smooth the transition.

## Supplementary Box 2. Cornelia de Lange syndrome emergency card

### HEALTH CARE PROFESSIONALS INFORMATION ABOUT CORNELIA DE LANGE SYNDROME (CdLS)

**General information**

Cornelia de Lange syndrome (CdLS) is characterized by intellectual disability, typical facial features, upper limb anomalies, growth disturbances, and a large variety of other signs and symptoms. It can be caused by pathogenic variants in one of six genes, the most common one being *NIPBL*.

**Main medical problems in CdLS**

- Short stature (specific growth charts available)
- Microcephaly
- Long term feeding difficulties
- Developmental delay/Intellectual disability
- Speech problems
- Behavioral problems, especially self-injurious behavior
- Severe recurrent gastro-esophageal reflux
- Constipation
- Small hands, missing fingers to absent fingers
- Hearing loss
- Ptosis, recurrent blepharitis, myopia
- Cryptorchidism
- Cutis marmorata; hirsutism

**Less frequent medical problems in CdLS**

- Heart malformations (ventricular septal defect, pulmonary stenosis)
- Diaphragmatic hernia
- Seizures
- Intestinal malrotation, duodenal atresia, annular pancreas
- Perthes disease, hip dislocations
- Scoliosis
- Barrett esophagus
- Renal malformations
- Immunological problems
- Dental crowding, caries
- Nystagmus, strabismus
- Cleft palate

**Acute life-threatening complications in CdLS**

- Bowel obstruction, volvulus
- Aspiration pneumonia (gastroesophageal reflux/swallowing difficulties)
- Seizures
- Cardiac problems
- Bladder infections
- Retinal detachment
- Small airways (anesthesia risk)

Further information can be obtained from CdLS World Federation www.cdlsworld.com

## Supplementary Box 3. Cornelia de Lange syndrome emergency card (Updated __/__/__)

**PERSONAL DETAILS**

Name ________________________________________

DOB _________________Gender________________

Address_______________________________________

Phone________________________________________

**PHYSICIAN DETAILS**

Name________________________________________

Phone________________________________________

Email_________________________________________

**EMERGENCY CONTACT**

Name___________________________________

Relation__________________________________

Phone___________________________________

Email____________________________________

Name___________________________________

Relation__________________________________

Phone___________________________________

Email____________________________________

**Typical vital parameters of patient**

Oxygen saturation (%) ___________________________

Heart rate (bpm) ________________________________

Blood pressure (mmHg) __________________________

Temperature regulation___________________________

Length/height (cm) _______________(___/___/_____)

Weight (Kg)_____________________(___/___/_____)

( ) NG tube ( ) G-tube type and size_____________

( ) Tracheostomy ( ) Mechanical ventilation

( ) Vascular access device __________________

**Allergies**

________________________________________________________________________________

**Major malformations**

( ) Cleft palate

( ) Genital anomalies: type______________________

( ) Intestinal malrotation; surgery no/ date__________

( ) Cardiac anomaly: type______________________

Last evaluation __/__/__ surgery no/ date________

( ) Upper limb malformation

**Psychomotor/cognitive development**

( ) Normal ( ) Borderline ( ) Disabled

Degree of delay: ( ) mild ( ) moderate

( ) severe ( ) profound

**Verbal communication**

( ) Absent ( ) Strongly limited ( ) Limited

( ) Near normal

**Behavioral problems**

( ) Anxiety ( ) Aggression ( ) Self-injurious

( ) Hyperactivity ( ) Autism spectrum disorder

Likes: ____________________________________

Dislikes: _________________________________

**Medical complications**

( ) Gastroesophageal reflux; surgery no/ date________

( ) Feeding problems

( ) Constipation: ( ) occasional ( ) often/frequent

( ) Food intolerance: ( ) Lactose ( ) Gluten

Other_____________ Special diet_______________

( ) Seizures: ( ) frequent ( ) rare Type____________

( ) medication: ___________________________

( ) Hearing loss: ( ) sensorineural ( ) conductive

( ) mild ( ) moderate ( ) severe ( ) hearing aids

( ) Visual impairment: type_____________ ( ) glasses

( ) Ptosis, surgery no/ date______________________

( ) Small airways

( ) Pneumonia (recurrent), dates_________________

( ) Ear infections (frequent) ( ) Sinus infections

( ) Renal problems: type________________________

( ) Hip problems: type__________________________

( ) Dental anomalies: ( ) cavities ( ) crowding

( ) allows inspection

( ) Other medical problems: type________________

**Medical treatment**

| Medication | Dosage | Frequency | Reason |
|---|---|---|---|
| | | | |
| | | | |
| | | | |
| | | | |
| | | | |
| | | | |

***Supplementary table 1.*** *Studies reporting level of intellectual disability in individuals with Cornelia de Lange syndrome.**

| Study | Participant characteristics | | | | Assessment | Individuals scoring within categories of intellectual disability n (%) | | | | | |
|---|---|---|---|---|---|---|---|---|---|---|---|
| | N | Age in years Mean (SD) range | % Male | Molecular confirmation | | Profound | Severe | Moderate | Mild | Borderline | Normal |
| Ajmone et al. (2014) | 17[1] | 8.2<br>2.5-13.4 | 47.1 | Yes | LIPS-R, GS | 2<br>(12%) | 0<br>(0%) | 4<br>(23%) | 1<br>(6%) | 2<br>(12%) | 3<br>(17%) |
| Basile et al. (2007) | 56[2] | 10.6 *(8.5)*<br>1-31 | 51.8 | No | LIPS-R, WS, SBIS; GS | 12<br>(21%) | 15<br>(27%) | 15<br>(27%) | 5<br>(9%) | 7<br>(12%) | 2<br>(4%) |
| Beck (1987) | 36 | Median: 16 | N/A | No | Unknown | 14<br>(39%) | 5<br>(14%) | 7<br>(19%) | 6<br>(17%) | 2<br>(6%) | 2<br>(6%) |
| Berney, Ireland & Burns (1999) | 49 | 10.2 *(7.8)* | 42.9 | No | Unknown | (43%) | (20%) | (18%) | (8%) | (10%) | --- |
| Kline et al. (2007) | 49 | 17.8<br>11–50 | 71.4 | 53% received molecular testing | Medical records | --- | (51%) | (24%) | (16%) | (6%) | (4%) |
| Olioso et al. (2009) | 45 | 22.4 | 51.1 | No | Unknown | --- | 24<br>(53%) | 17<br>(38%) | 4<br>(9%) | --- | --- |
| Sarimski (1997) | 27 | 7.1 (4.9)<br>1–16 | 44.4 | No | Estimated from parental report | --- | 19<br>(70%) | 8<br>(30%) | --- | --- | --- |
| | | | | | | **Mean (*SD*); range** | | | | | |
| Fraser et al., (1978) | 6[3] | 14-22 | 100.0 | No | SBIS; LIPS | IQ = <30–54) | | | | | |
| Kline et al. (1993) | 14 | 3.2 – 19.0 | N/A | No | SBIS; MSCA; WISC-R; WPPSI-R | IQ = 53; 30–85 | | | | | |
| Lorusso et al. (2007) | 6 | 14.8 *(13.1)* | 16.7 | No | WPPSI, WISC-R, WAIS-R | IQ = 63.8 (*17.7*) 47–95 | | | | | |
| Moeschler & Graham (1993) | 3 | 0.8-13 | 100.0 | No | Unknown; SBIS; WISC | IQ = 63–66 | | | | | |
| Parisi, Di Filippo & Roccella (2015) | 4 | 5.25<br>4–5 | 75.0 | No | WPPSI | VIQ = 52.8; 45–58. PIQ = 61.3; 51–66 | | | | | |
| Huisman (2017) | 39 | Median13<br>0-46 | 27.5 | Yes,SMC1A | Physician report | 1/20<br>(5%) | 5/20<br>(25%) | 8/20<br>(40%) | 4/20<br>(20%) | --- | 2/20<br>(10%) |
| | 67 | Median 14<br>0-46 | 50.7 | Yes, NIPBL | Physician report | 11/58<br>(19%) | 27/58<br>(47%) | 16/58<br>(28%) | 4/58<br>(7%) | --- | 0/58<br>(0%) |

*Inclusion criteria for studies varied and may explain in part differences in scoring results
[1] 5 participants were unable to be assessed for reasons not given.
[2] 8 participants were unable to be formally assessed due to level of disability.
[3] 3 participants were unable to be assessed for reasons not given

**VIQ =** Verbal IQ**; PIQ =** Performance IQ; **GS** = Griffiths' Scale; **LIPS-R** = Leiter International Performance Scales-Revised; **MSCA** = McCarthy Scales of Children's Abilities; **SBIS** = The Standford-Binet Intelligence Scale; **WS =** Welchsler Scales); **WAIS-R** = Wechsler Adult Intelligence Scale-Revised; **WISC-R** = Wechsler Intelligence Scale for Children – Revised; **WPPSI** = Wechsler Preschool Performance Scale of Intelligence; **WPPSI-R** = Wechsler Preschool Performance Scale of Intelligence - Revised.

***Supplementary table 2.*** *Studies reporting level of developmental delay in individuals with Cornelia de Lange syndrome.**

| Study | Participant characteristics | | | | Assessment | Individuals scoring within categories of developmental delay n (%) | | | | | |
|---|---|---|---|---|---|---|---|---|---|---|---|
| | **N** | **Age in years Mean (SD) range** | **% Male** | **Molecular confirmation** | | **Profound** | **Severe** | **Moderate** | **Mild** | **Borderline** | **Normal** |
| Bhuiyan et al. (2006) | 36 | N/A | N/A | Yes | VABS | 19 (53%) | 6 (16.5%) | 6 (16.5%) | 4 (11%) | 1 (3%) | --- |
| Marchisio et al. (2008) | 50 | Median: 6.5 1-18 | 46.0 | No | Categorised based on language and cognition | --- | 20 (40%) | 24 (48%) | 6 (12%) | --- | --- |
| Moss et al. (2008) | 34 | 12.4 *(3.8)* 5-18.96 | 47.2 | No | VABS | 9 (26%) | 16 (47%) | 6 (18%) | 3 (9%) | 0 (0%) | --- |
| Nakanishi et al. (2013) | 66 | 14.7 4 – 44 | 47.0 | No | VABS | 9 (14%) | 3 (4%) | 9 (14%) | 35 (53%) | 10 (15%) | --- |
| Oliver et al. (2009) | 54 | 13.9 *(9.0)* | 46 | No | VABS | 27 (50%) | 13 (24%) | 8 (15%) | 6 (11%) | --- | --- |
| Richards et al. (2009) | 12 | 11.0 *(5.2)* 5.0-18.0 | 33.3 | No | VABS | --- | 4 (33%) | 5 (42%) | 3 (25%) | --- | --- |
| Selicorni et al. (2007) | 62 | 12.0 0.5-48.0 | 61.3 | Yes | Unknown | 16 (26%) | 0 (0%) | 36 (58%) | 5 (8%) | --- | --- |
| Wulffaert et al. (2009) | 37 | 18.1 *(13.0)* 1.4 – 46.2 | 56.8 | Yes | VABS | 19 (51%) | 6 (16%) | 6 (16%) | 5 (14%) | 1 (3%) | --- |
| Yan et al. (2006) | 28 | 10.9 3–27 | 53.6 | Yes | Based on criteria from Gillis et al. (2004) | --- | 15 (54%) | 9 (32%) | 4 (14%) | --- | --- |
| | | | | | | **Mean (*SD*); range** | | | | | |
| Basile et al. (2007) | 56 | 10.6 *(8.5)* 1-31 | 51.8 | No | VABS | AE (years) = 3.5 (*2.9*) 1.5–11 | | | | | |
| Crawford et al. (2015) | 15 | 18.4 *(9.8)* 6.7-33.4 | 53.3 | No | VABS | SS = 60 (*25)*; 21–121 | | | | | |
| Kline et al. (1993) | 36 | 3.2 – 19.0 | N/A | No | VABS | SS = 48; 20–87 | | | | | |
| Moss et al. (2005) | 8 | 9.8 4.3-14.3 | 62.5 | No | VABS | AE (months) = 25; 6–47 | | | | | |
| Oliver et al. (2006) | 16 | 7.6 1.7-16.1 | 56.3 | No | VABS | AE (months) = 11.6 *(4.9)*; 4–23 | | | | | |
| Srivastava et al. (2014) | 41 | 11.4 *(3.8)* 5–18 | 43.9 | No | VABS | SS = 38.3 (*23.1*) | | | | | |

**AE** = Age Equivalence**; SS** = Standard Score; **VABS**= Vineland Adaptive Behavior Scales ; *Inclusion criteria for studies varied and may explain in part differences in scoring result
